# Supplementary figures and images for: A modular framework for multiscale, multicellular, spatiotemporal modeling of acute primary viral infection and immune response in epithelial tissues and its application to drug therapy timing and effectiveness
Source: PLoS Comput Biol. 2020 Dec 21;16(12):e1008451. doi: 10.1371/journal.pcbi.1008451 (PMC7785254; doi:10.1371/journal.pcbi.1008451)

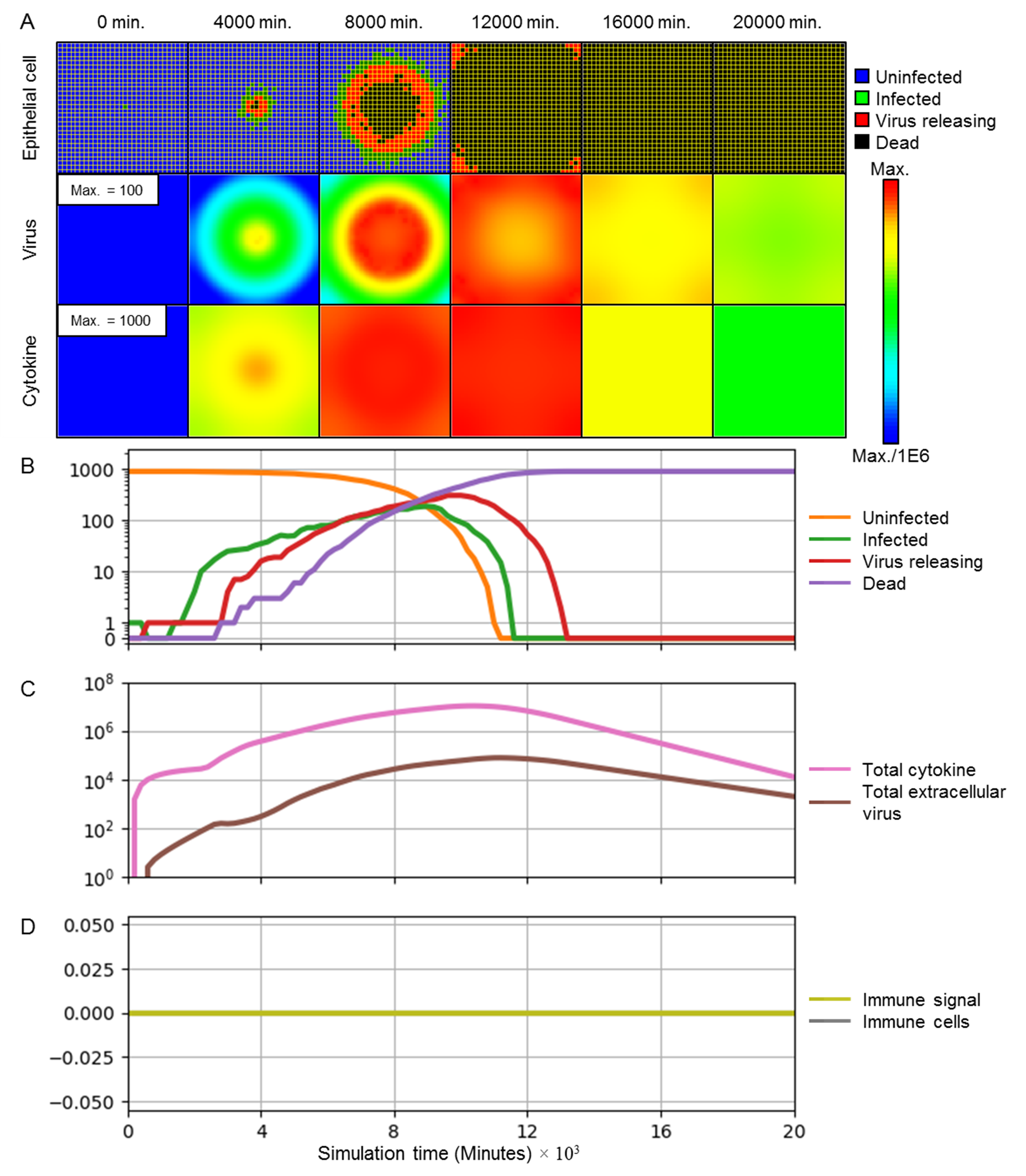

Supplement: S1 Fig — Simulation of the progression of infection in a patch of epithelial tissue, with all parameters as in Fig 3, but with no cellular immune system response corresponding to virus spread in an in vitro or organoid culture, or a severely immunosuppressed individual. (A) Snapshots of spatial configuration vs time showing progression of simulated infection. Columns, left to right: 0 minutes (time of initial infection), 4000 minutes (67 hours, 2 ¾ days) after infection, 8000 minutes (133 hours, 5 ½ days), 12000 minutes (200 hours, 8 ⅓ days), 16000 minutes (267 hours, 11 days), and 20000 minutes (333 hours, 14 days). First row: epithelial cell layer composed of uninfected (blue), infected (green), virus releasing (red) cells and dead cells (black). Second row: level of extracellular virus field. Third row: extracellular cytokine field. Fields are color-coded on a logarithmic scale: red corresponds to the chosen maximum value specified in the first panel, blue to six orders of magnitude lower than the maximum value, and values outside this range are colored as their closest border value. (B-D) Simulation time series. (B) Number of uninfected (orange), infected (green), virus releasing (red) and dead (purple) epithelial cells on a logarithmic scale vs time vs time in minutes. (C) Total extracellular cytokine (magenta) and total extracellular virus (brown) on a logarithmic scale vs time in minutes. (D) Value of the immune recruitment signal S (yellow) and number of immune cells (grey) on a linear scale vs time in minutes. (TIF) [file pcbi.1008451.s001.tif]

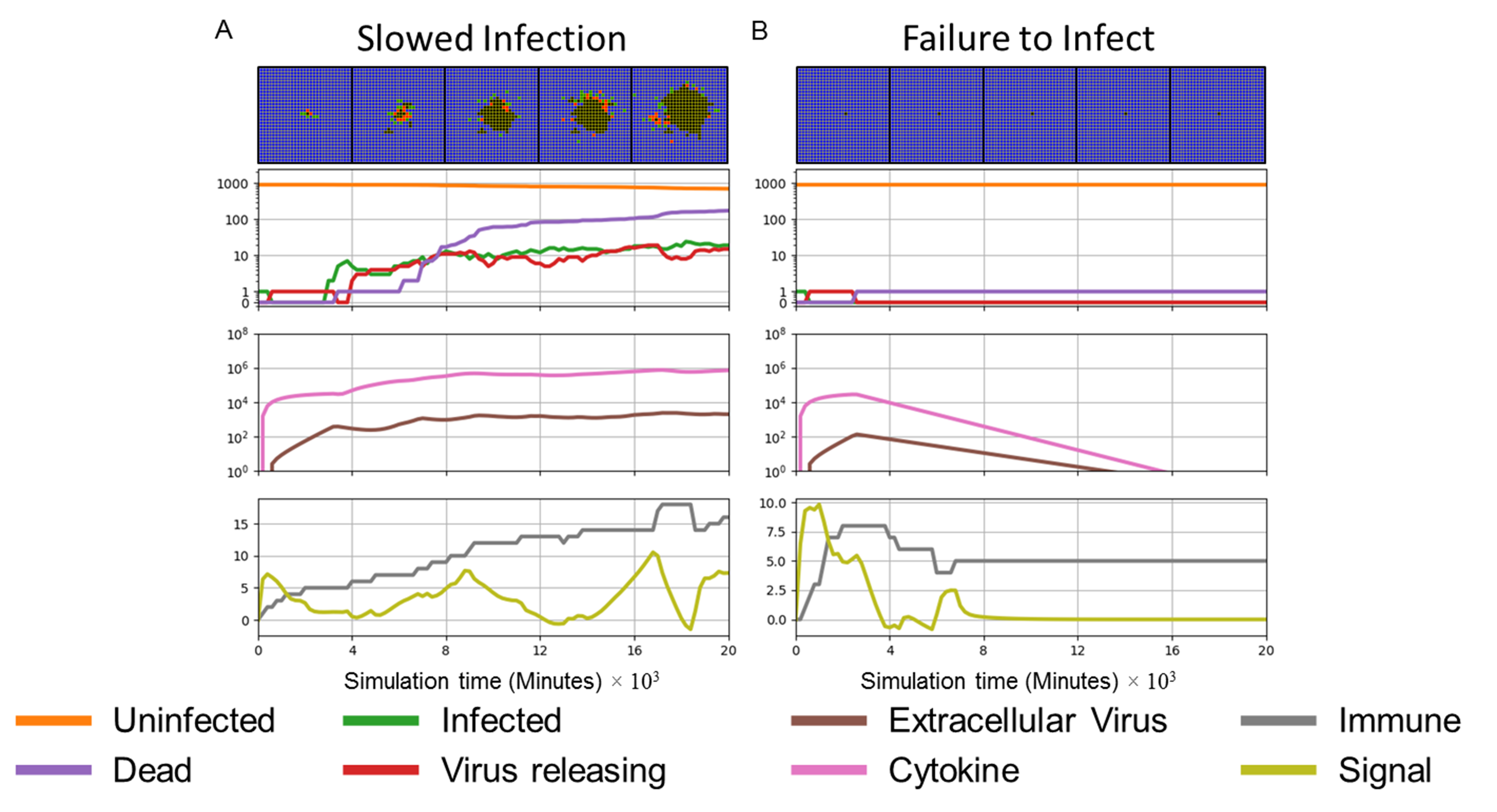

Supplement: S2 Fig — (A) A border case of slowed infection towards containment is Slowed Infection with constant virus: when the net effectiveness of viral and immune dynamics are balanced, the number of infected cells and the total extracellular virus fluctuate around steady state levels. (B) A limit case of Clearance is the failure to infect: initially infected cells may replicate and secrete virus, but insufficiently so to infect other cells during simulation time such that any initially infected cells vanish and total extracellular virus drops below a threshold of 10−3 per cell area. (TIF) [file pcbi.1008451.s002.tif]

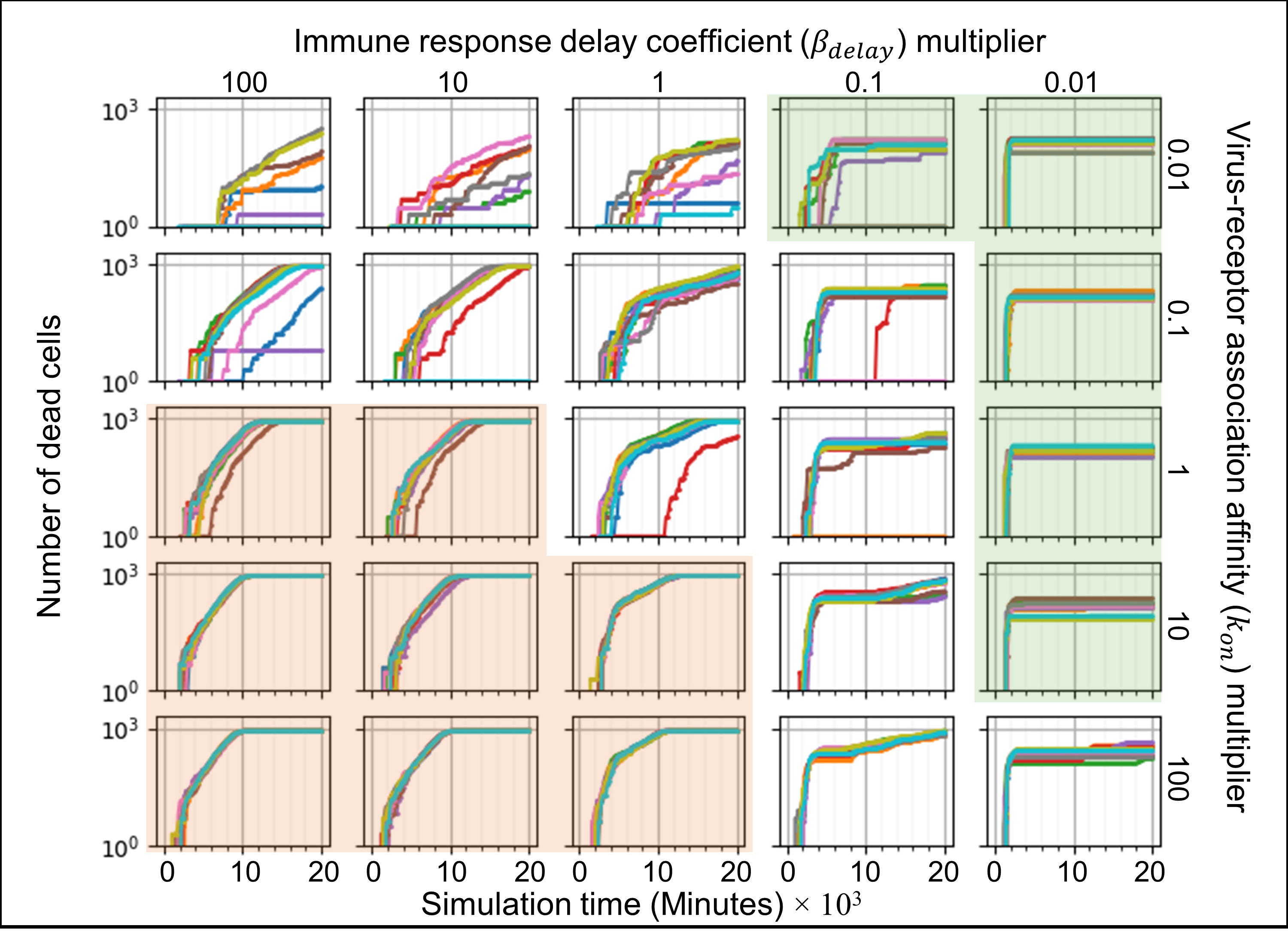

Supplement: S3 Fig — Logarithmic multidimensional parameter sweep performed by running 10 simulation replicas increasing and decreasing the baseline parameter values 10-fold and 100-fold for all parameter sets and replicas in Fig 5. Results show consistent containment/clearance for small kon and small βdelay (upper right, green-shaded subplots), widespread infection for high kon and small βdelay (lower left, orange-shaded subplots), and multiple outcomes for the same parameter values (uncolored subplots). Number of cells are shown on a logarithmic scale vs time in minutes. (TIF) [file pcbi.1008451.s003.tif]

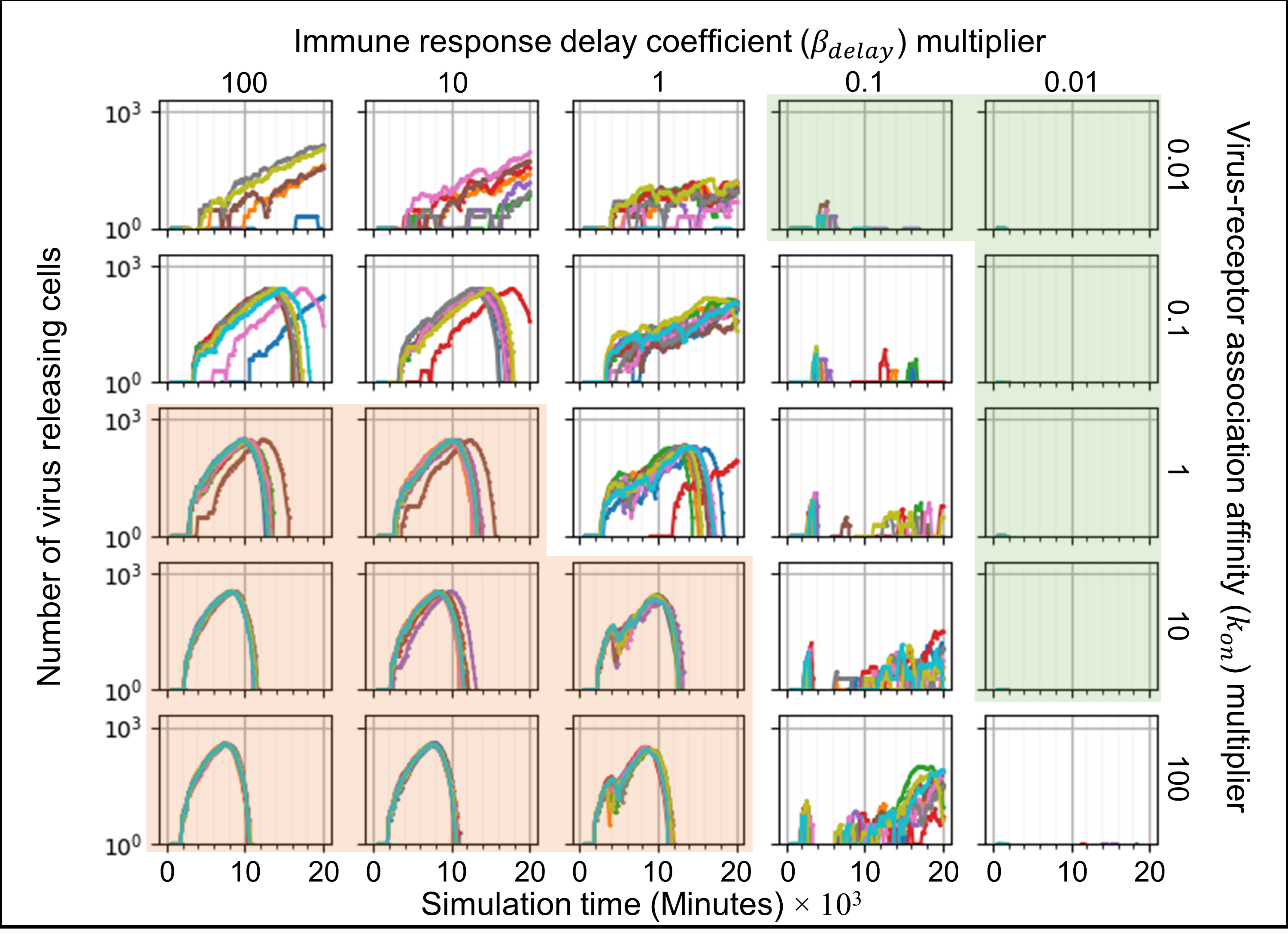

Supplement: S4 Fig — Logarithmic multidimensional parameter sweep performed by running 10 simulation replicas increasing and decreasing the baseline parameter values 10-fold and 100-fold for all parameter sets and replicas in Fig 5. Results show consistent containment/clearance for small kon and small βdelay (upper right, green-shaded subplots), widespread infection for high kon and small βdelay (lower left, orange-shaded subplots), and multiple outcomes for the same parameter values (uncolored subplots). Number of cells are shown on a logarithmic scale vs time in minutes. (TIF) [file pcbi.1008451.s004.tif]

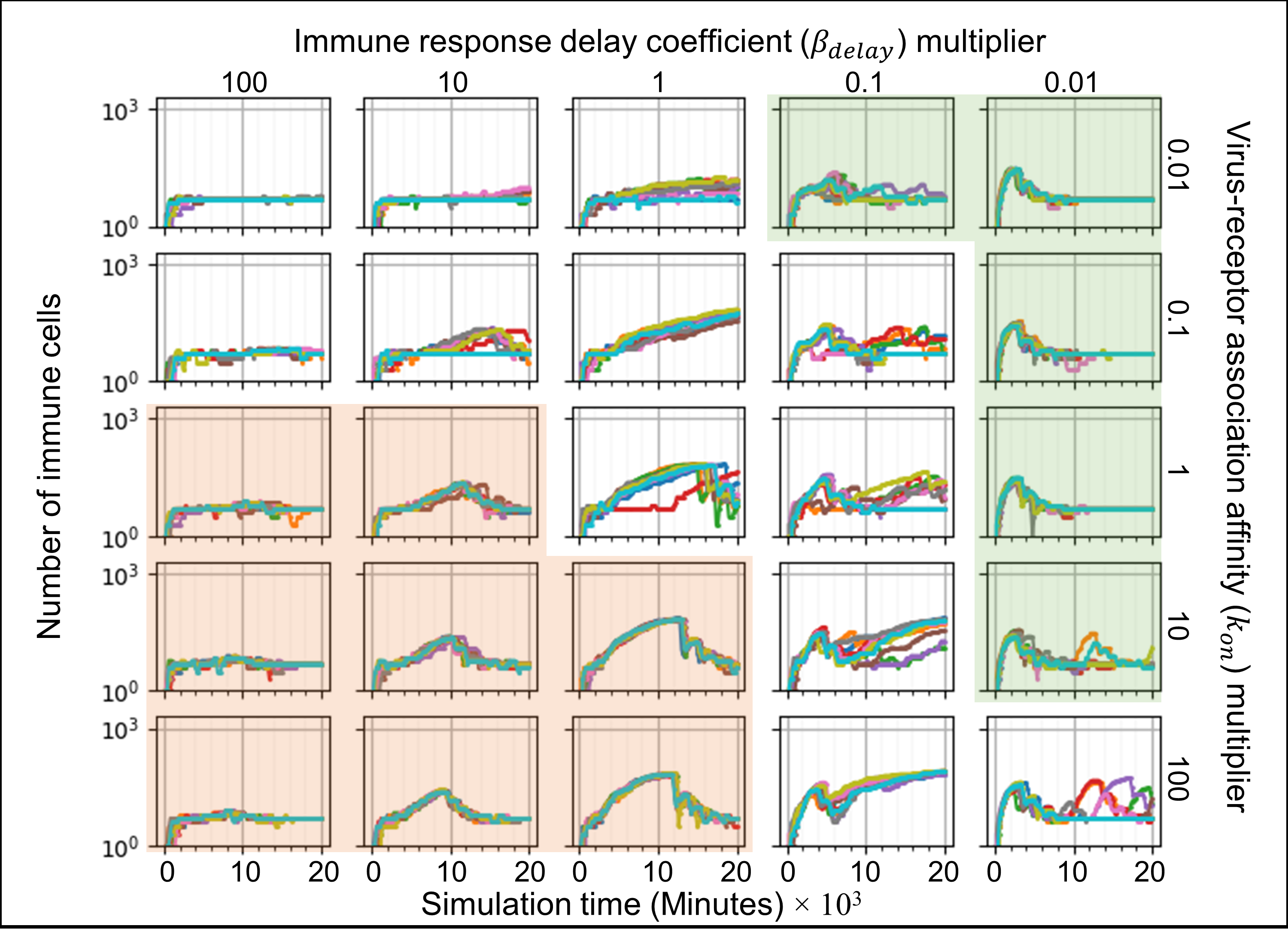

Supplement: S5 Fig — Logarithmic multidimensional parameter sweep performed by running 10 simulation replicas increasing and decreasing the baseline parameter values 10-fold and 100-fold for all parameter sets and replicas in Fig 5. Results show consistent containment/clearance for small kon and small βdelay (upper right, green-shaded subplots), widespread infection for high kon and small βdelay (lower left, orange-shaded subplots), and multiple outcomes for the same parameter values (uncolored subplots). Number of cells are shown on a logarithmic scale vs time in minutes. (TIF) [file pcbi.1008451.s005.tif]

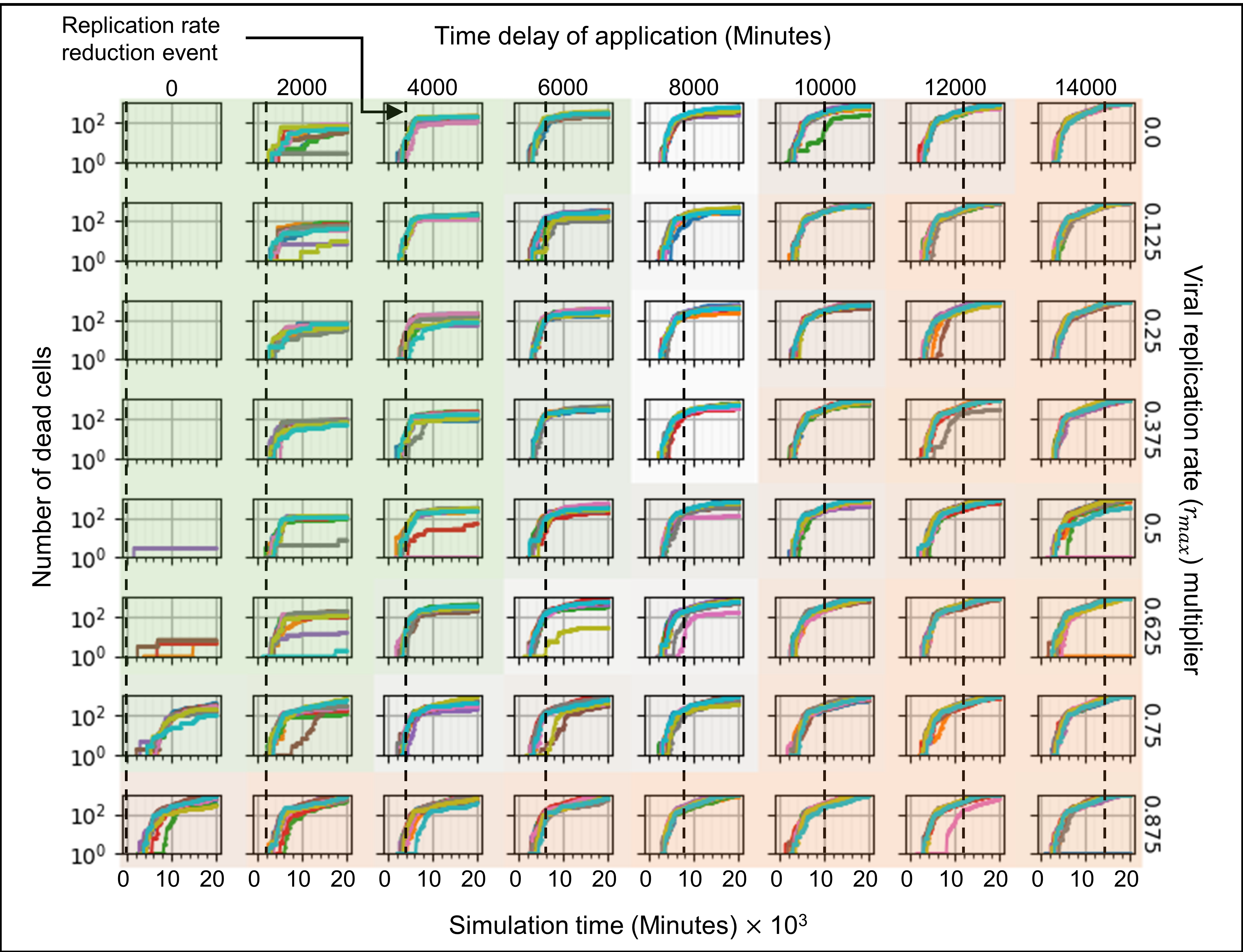

Supplement: S7 Fig — Time series of the number of dead cells for each simulation replica in Fig 8. Number of cells is shown on a logarithmic scale vs time in minutes. (TIF) [file pcbi.1008451.s007.tif]

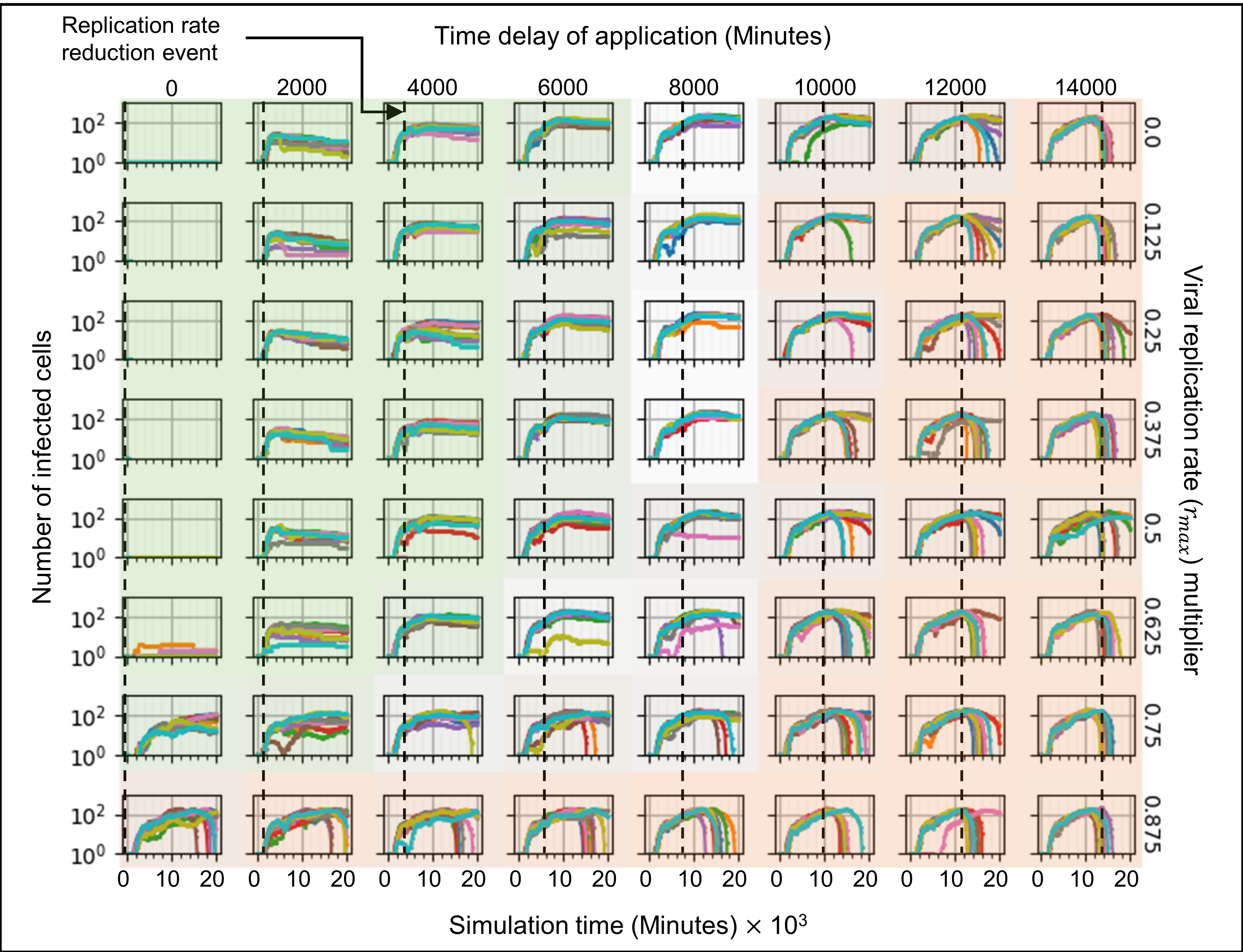

Supplement: S8 Fig — Time series of the number of infected cells for each simulation replica in Fig 8. Number of cells is shown on a logarithmic scale vs time in minutes. (TIF) [file pcbi.1008451.s008.tif]

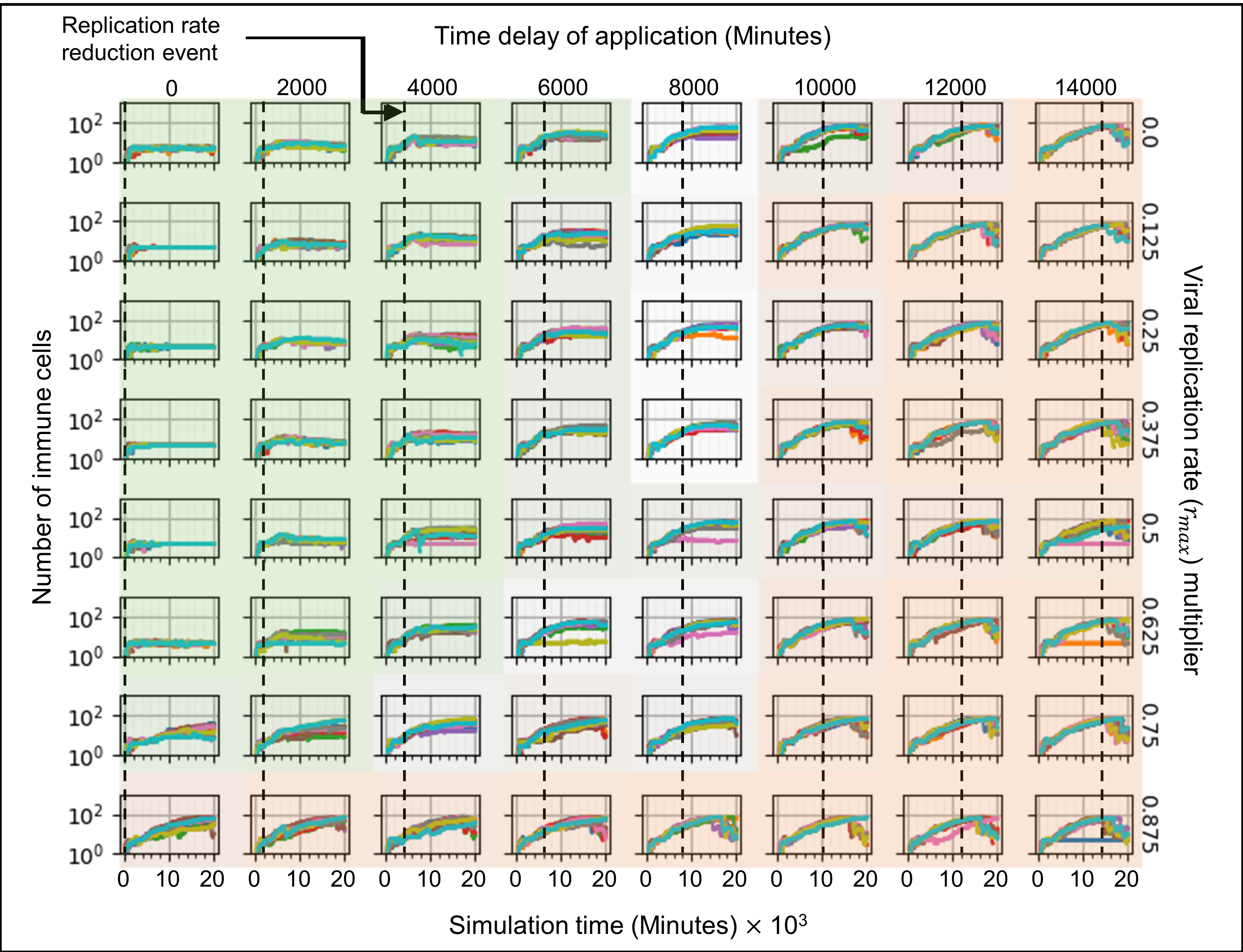

Supplement: S9 Fig — Time series of the number of immune cells for each simulation replica in Fig 8. Number of cells is shown on a logarithmic scale vs time in minutes. (TIF) [file pcbi.1008451.s009.tif]

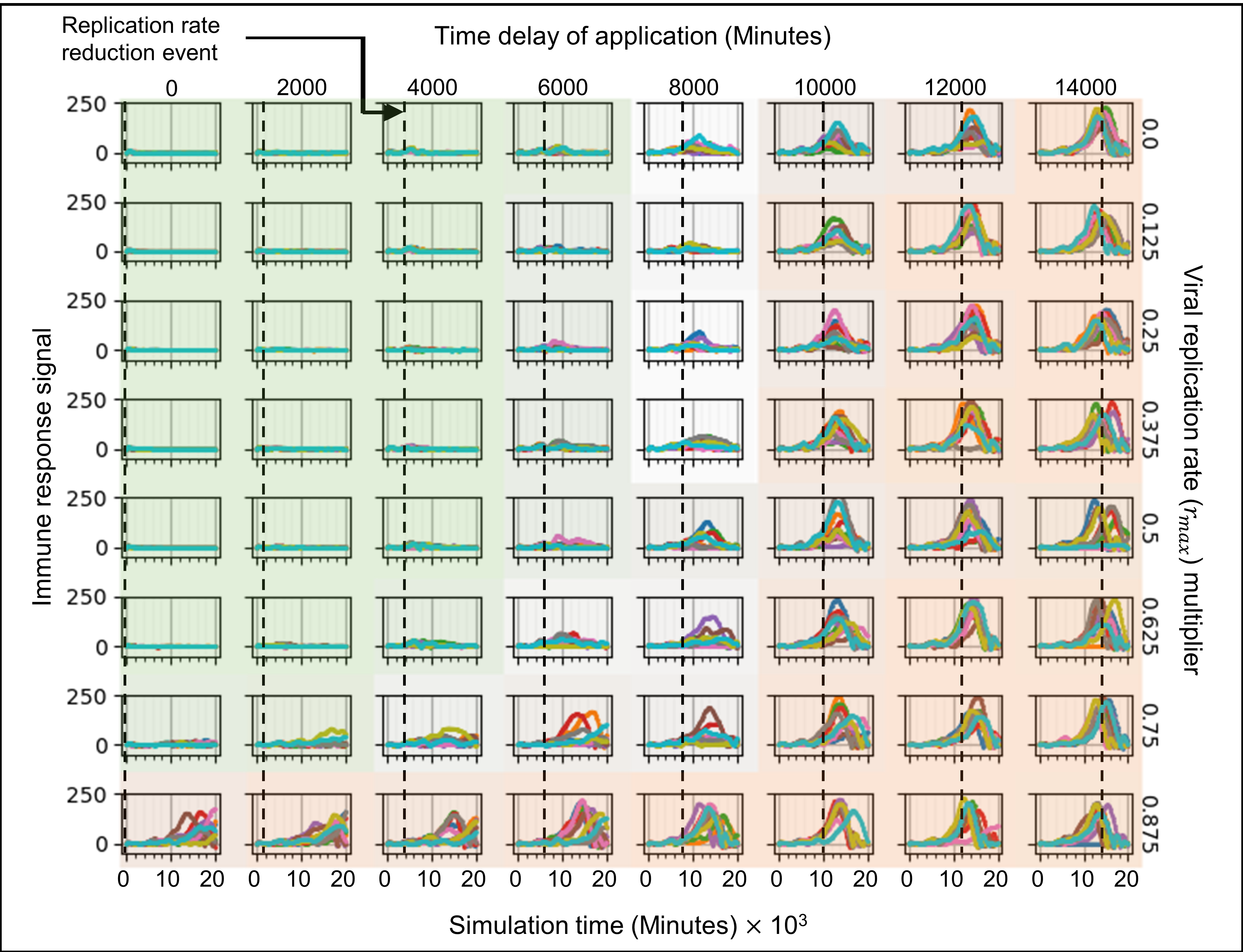

Supplement: S10 Fig — Time series of the immune response state variable S for each simulation replica in Fig 8. S is shown on a linear scale vs time in minutes. (TIF) [file pcbi.1008451.s010.tif]

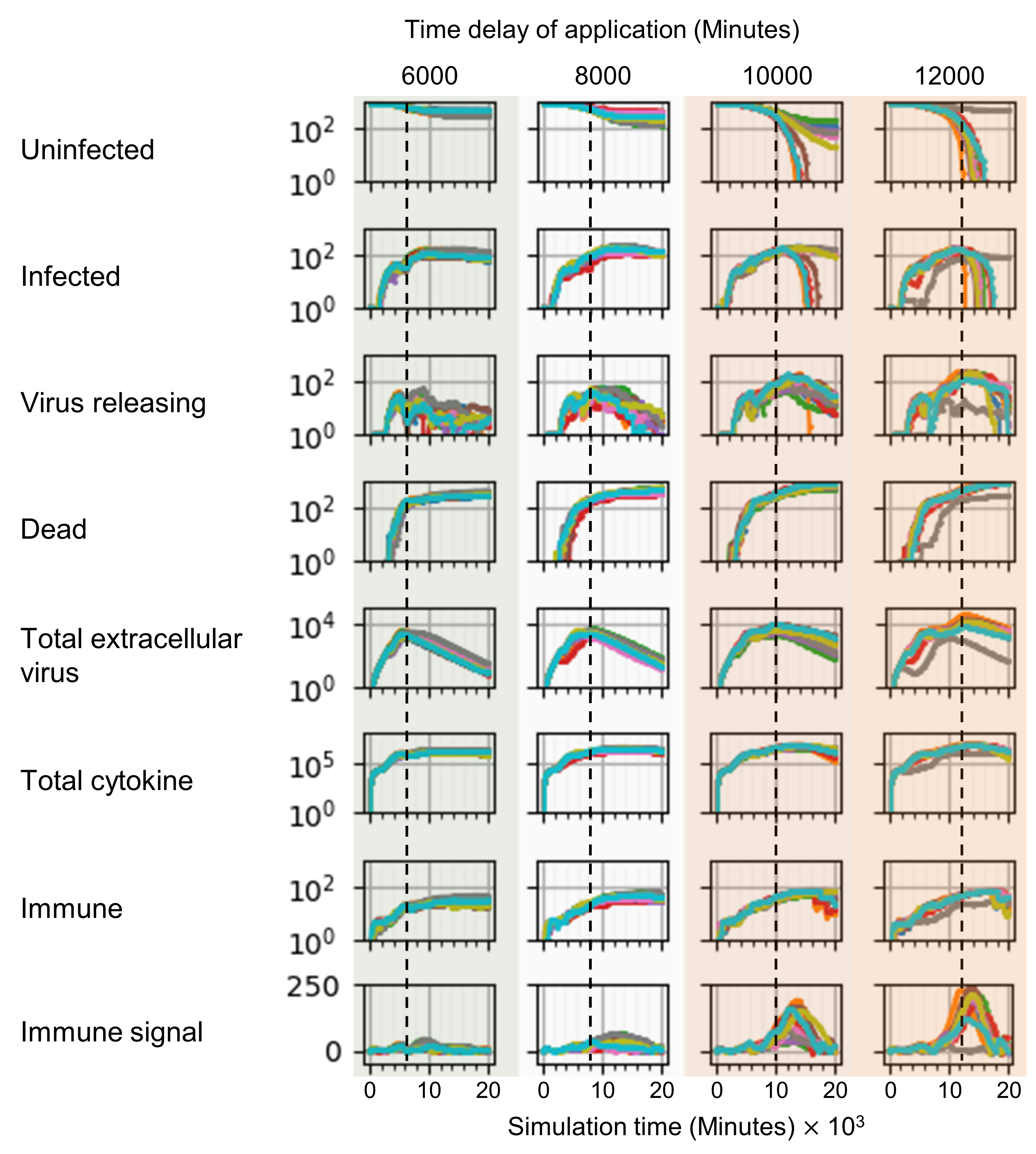

Supplement: S11 Fig — Simulations and parameters are as in Figs 9–11, for a viral replication rate multiplier of 0.375 and, from left to right, time delays of application of 6000, 8000, 10000, and 12000 minutes (100, 133, 167 and 200 hours, 4, 5 ½, 7 and 8 ⅓ days) (dashed lines). Results from all simulation replicas are shown vs time in minutes for, from top to bottom: number of uninfected cells, number of infected cells, number of virus releasing cells, number of dead cells, total extracellular virus, total cytokine, number of immune cells, and immune response state variable S. Parameter set subplots are shaded as in Figs 9–11 according to simulation outcomes. (TIF) [file pcbi.1008451.s011.tif]

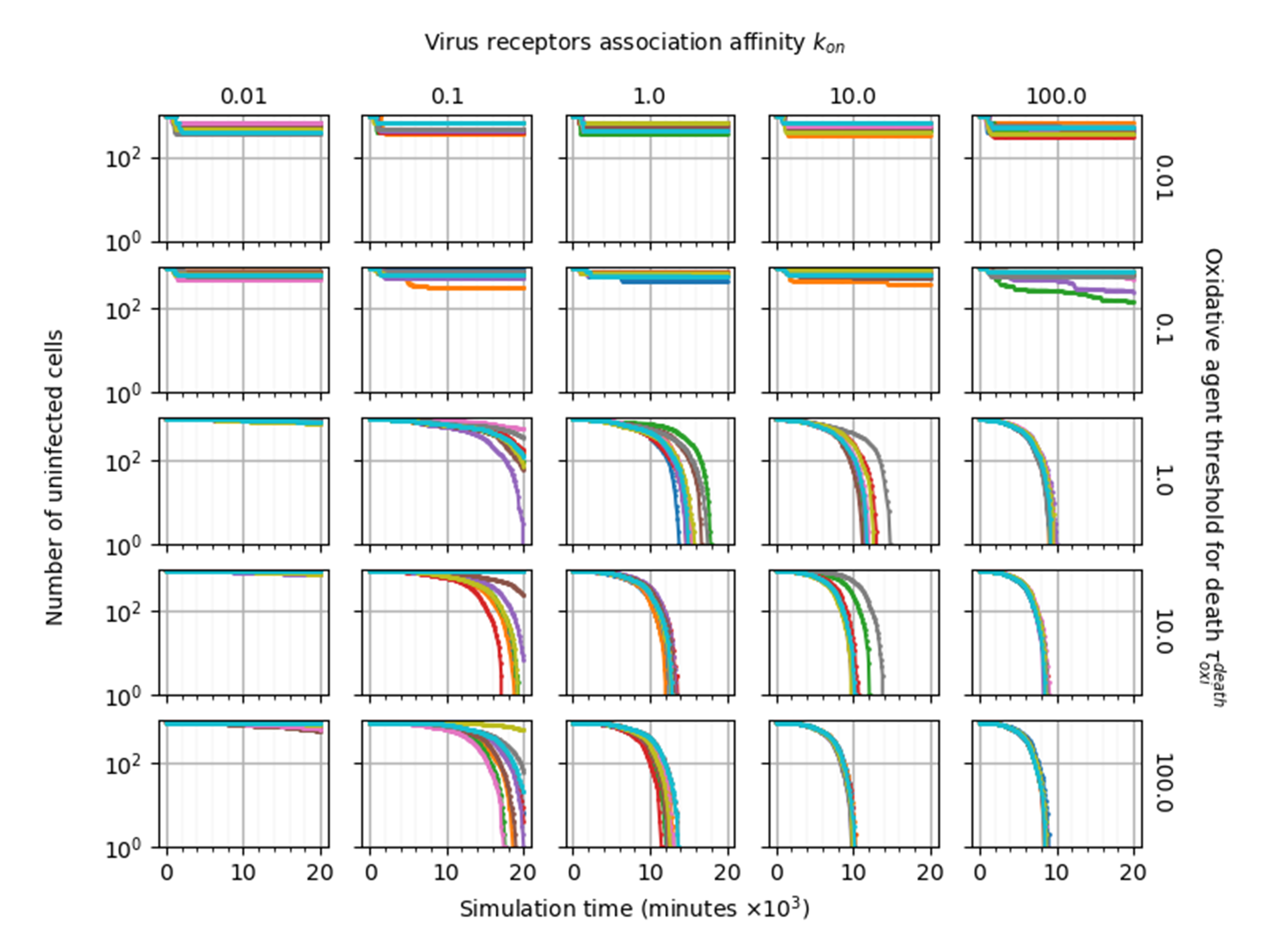

Supplement: S12 Fig — The number of uninfected epithelial cells for each simulation replica for each parameter set, plotted on a logarithmic scale, vs time displayed in minutes. (TIF) [file pcbi.1008451.s012.tif]

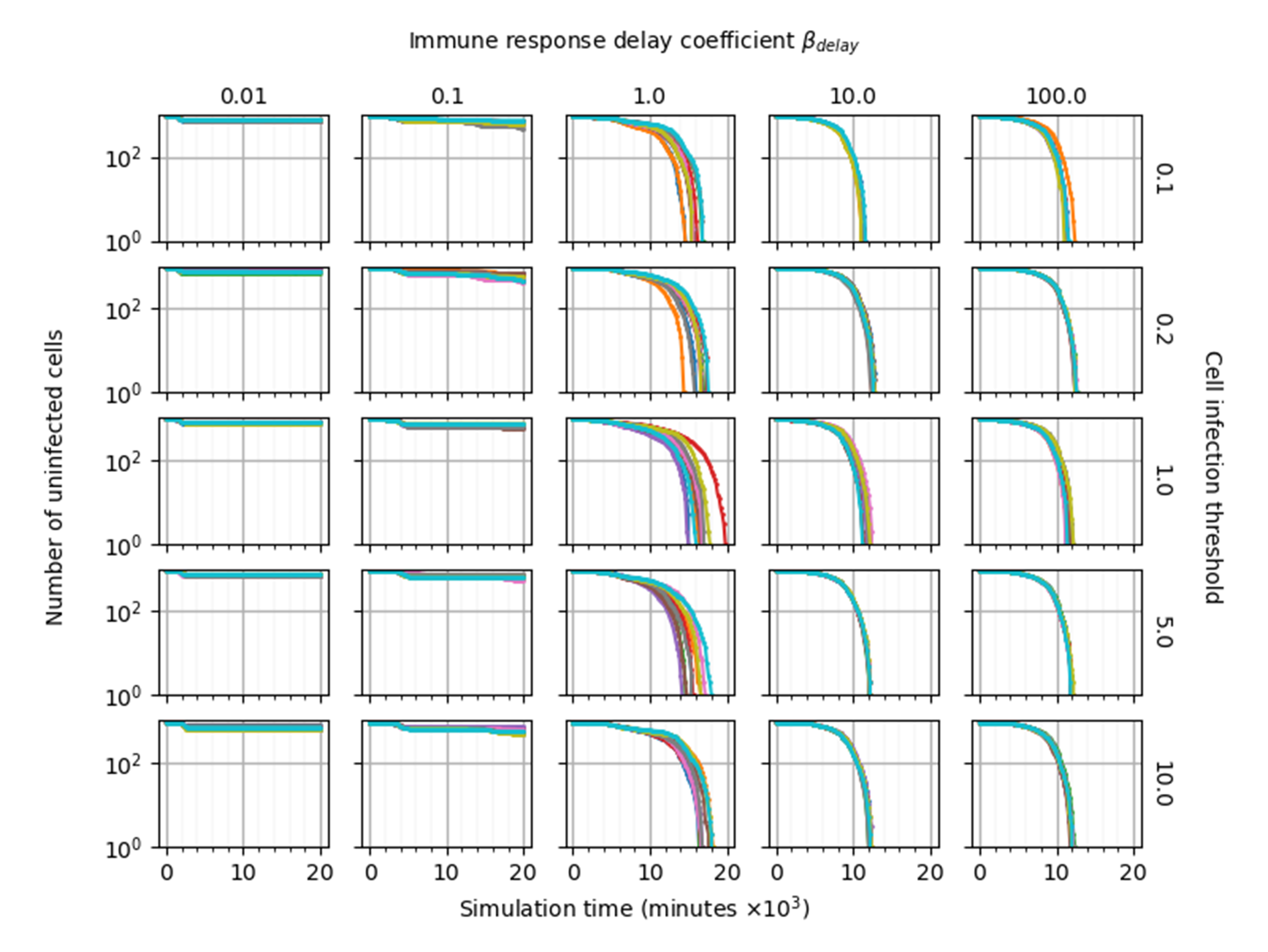

Supplement: S13 Fig — The number of uninfected epithelial cells for each simulation replica for each parameter set, plotted on a logarithmic scale, vs time displayed in minutes. (TIF) [file pcbi.1008451.s013.tif]

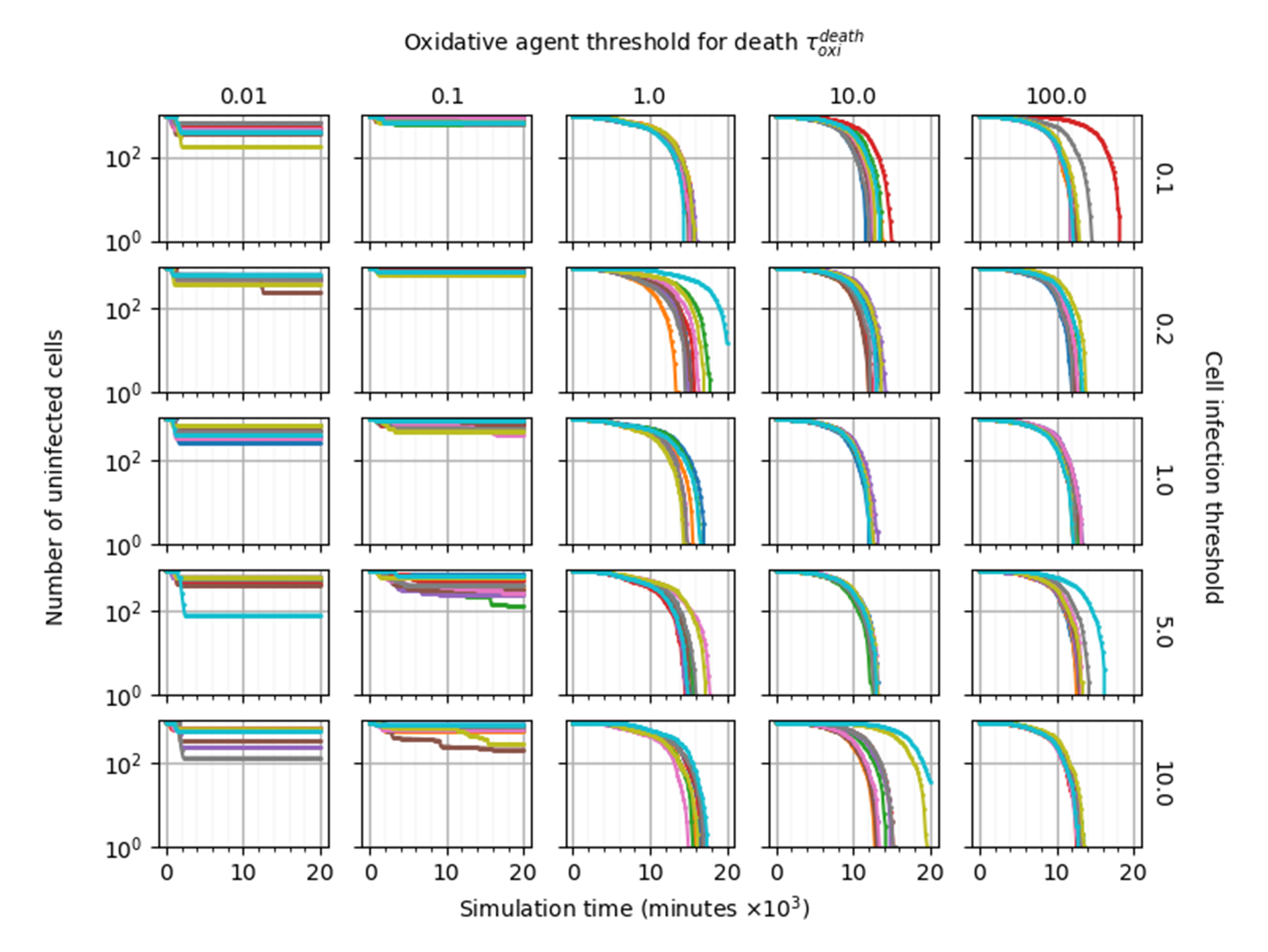

Supplement: S14 Fig — The number of uninfected epithelial cells for each simulation replica for each parameter set, plotted on a logarithmic scale, vs time displayed in minutes. (TIF) [file pcbi.1008451.s014.tif]

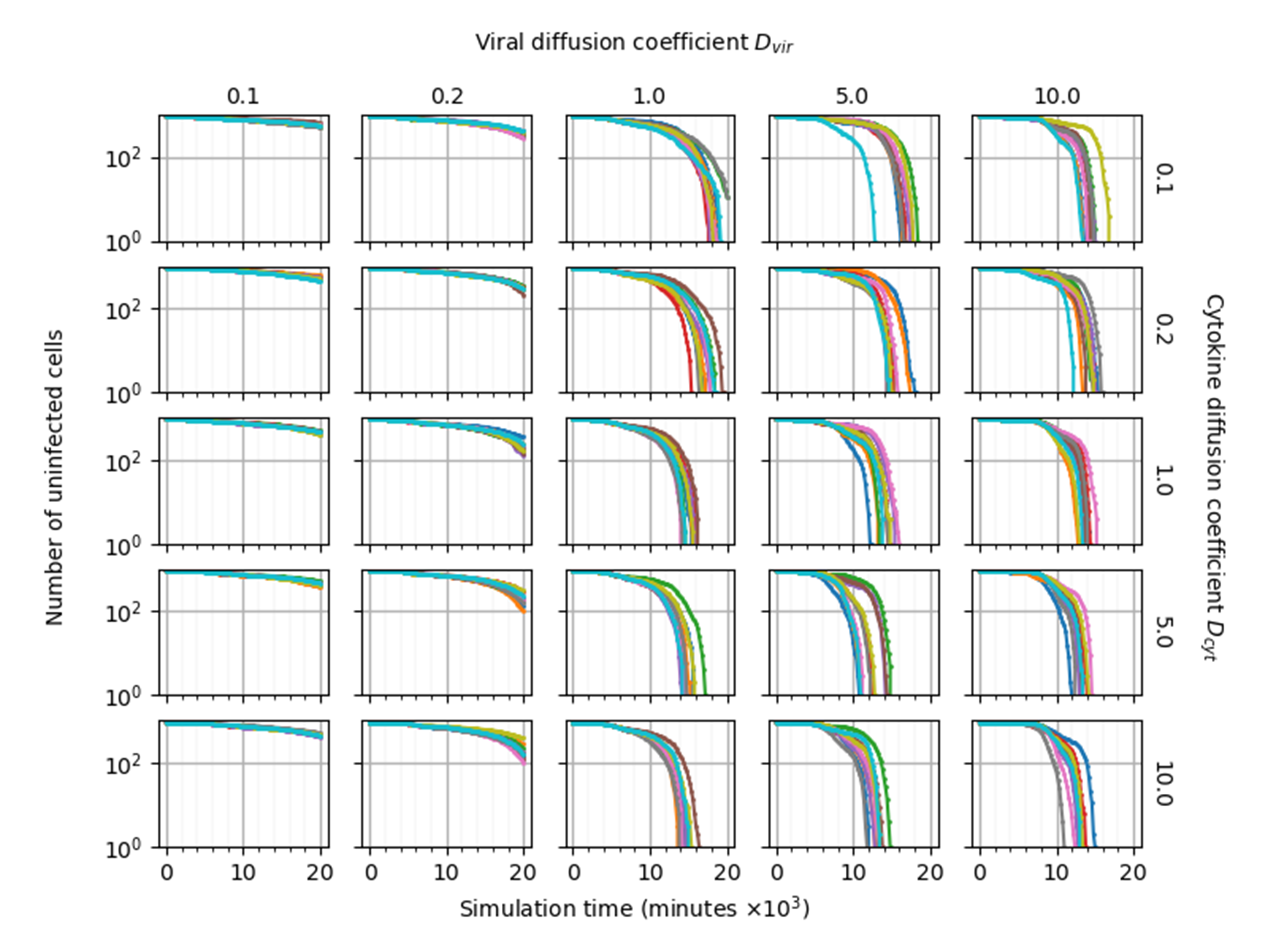

Supplement: S15 Fig — The number of uninfected epithelial cells for each simulation replica for each parameter set, plotted on a logarithmic scale, vs time displayed in minutes. (TIF) [file pcbi.1008451.s015.tif]

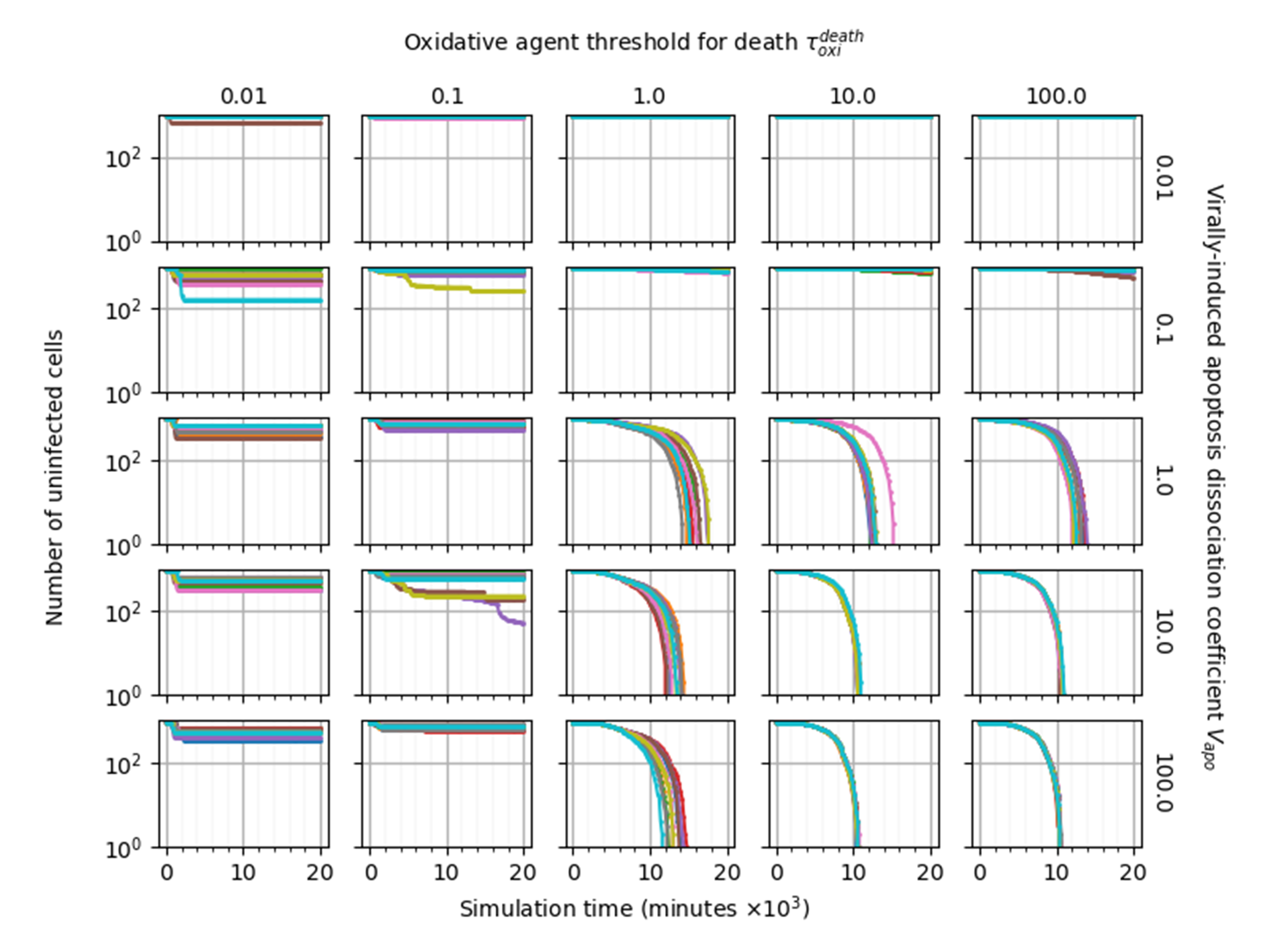

Supplement: S16 Fig — The number of uninfected epithelial cells for each simulation replica for each parameter set, plotted on a logarithmic scale, vs time displayed in minutes. (TIF) [file pcbi.1008451.s016.tif]

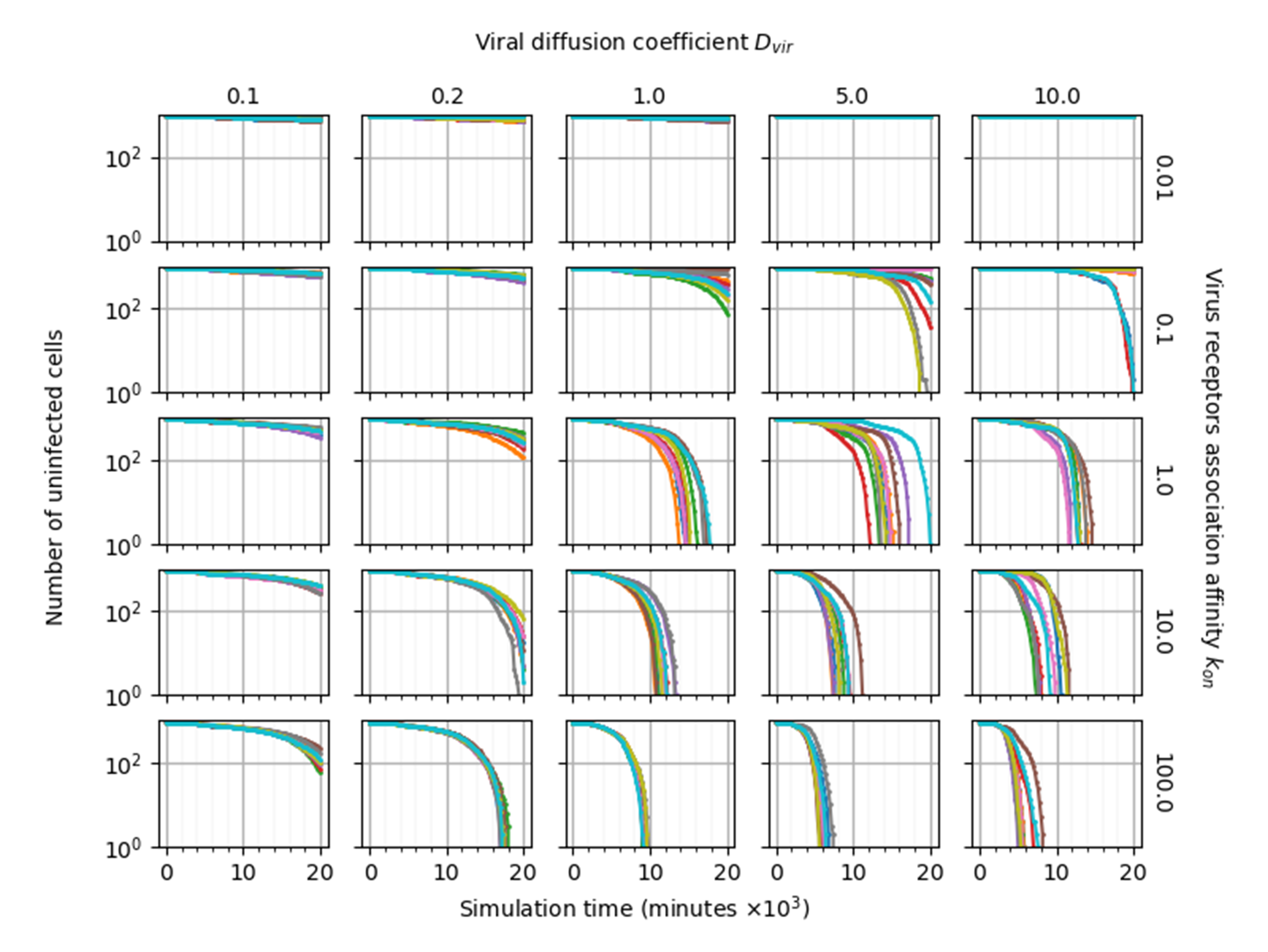

Supplement: S17 Fig — The number of uninfected epithelial cells for each simulation replica for each parameter set, plotted on a logarithmic scale, vs time displayed in minutes. (TIF) [file pcbi.1008451.s017.tif]

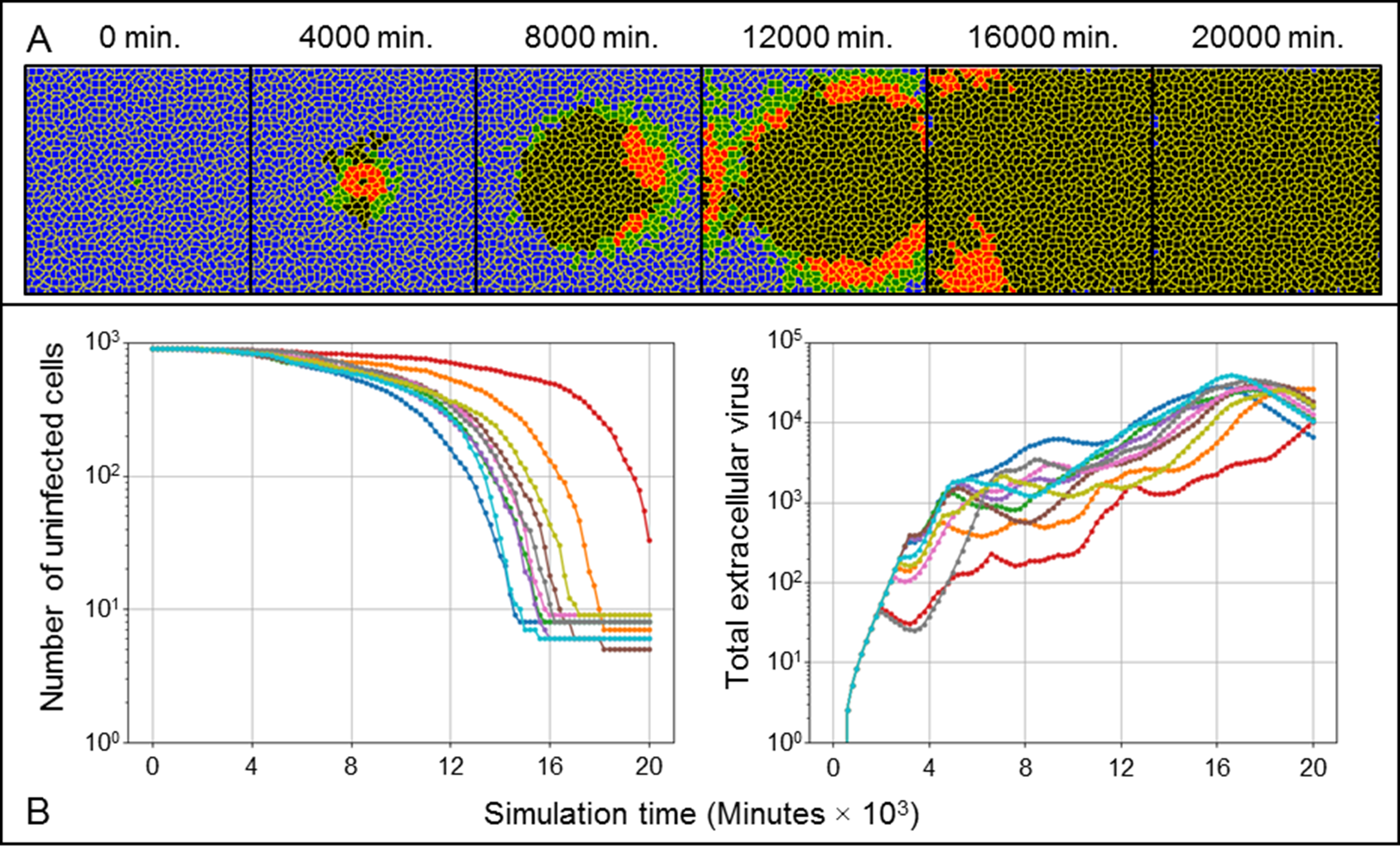

Supplement: S18 Fig — (A) Widespread infection occurs with the same spatiotemporal features as in Fig 3A in a non-uniform epithelial sheet. (B) Ten simulation replicas with a non-uniform epithelial sheet showed no significant differences in transient metrics compared to simulations with a uniform epithelial sheet. (TIF) [file pcbi.1008451.s018.tif]

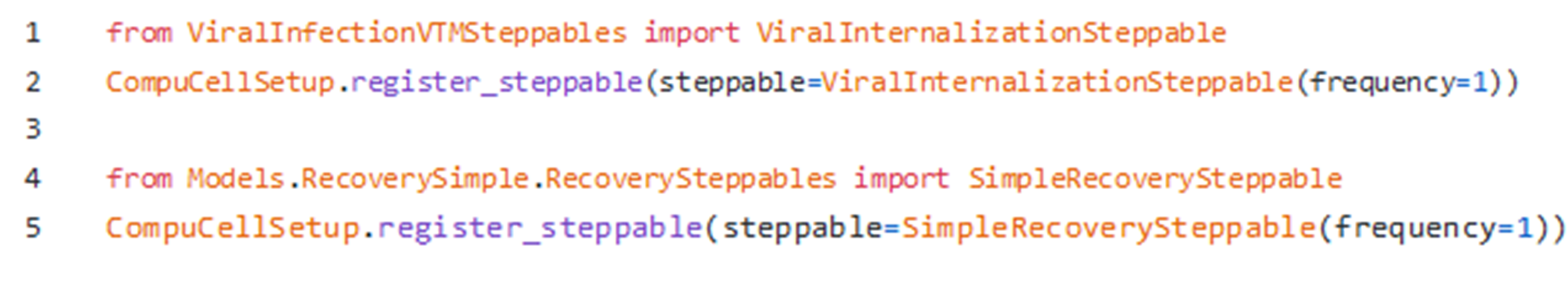

Supplement: S1 Code Snippet — (TIF) [file pcbi.1008451.s021.tif]

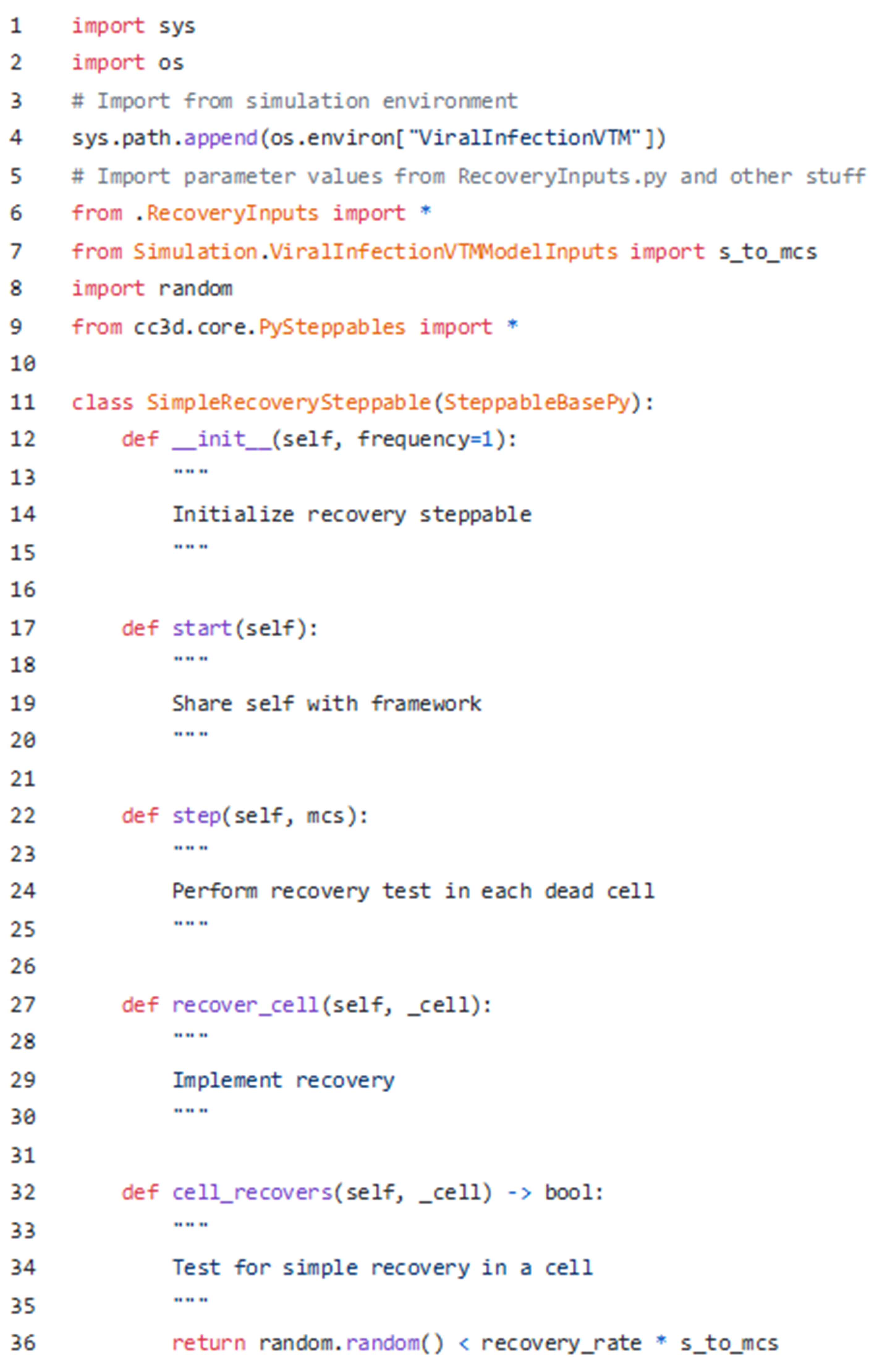

Supplement: S2 Code Snippet — The exact code of the implementation is shown for the steppable function “cell_recovers” (Lines 32–36). (TIF) [file pcbi.1008451.s022.tif]

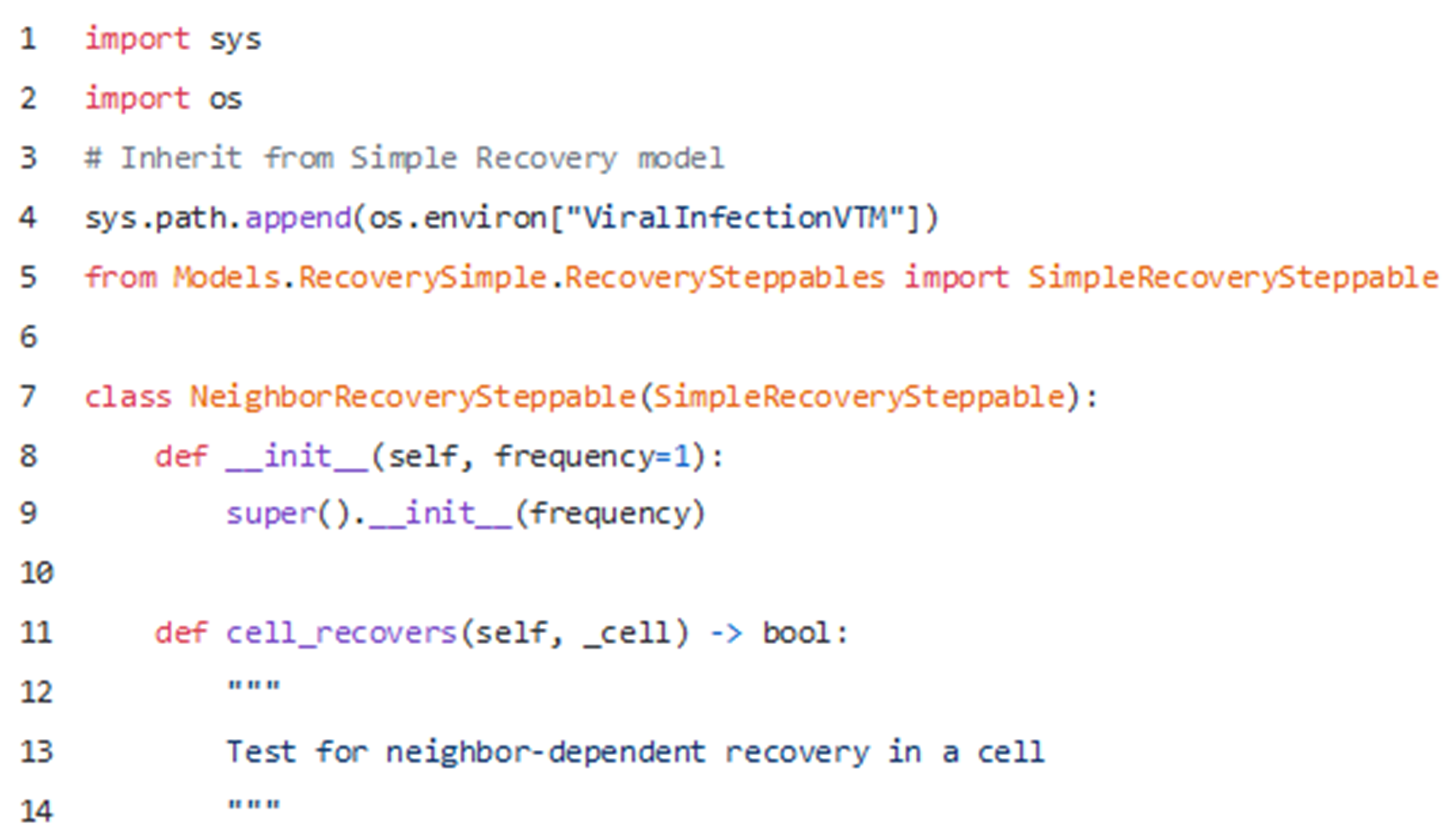

Supplement: S3 Code Snippet — (TIF) [file pcbi.1008451.s023.tif]
