# Supplementary material for: A modular framework for multiscale, multicellular, spatiotemporal modeling of acute primary viral infection and immune response in epithelial tissues and its application to drug therapy timing and effectiveness
Source: PLoS Comput Biol. 2020 Dec 21;16(12):e1008451. doi: 10.1371/journal.pcbi.1008451 (PMC7785254; doi:10.1371/journal.pcbi.1008451)
Supplement: S1 Table — Virus-receptor association affinity and immune response delay coefficient shown for no immune response (Fig 4A), widespread infection (Fig 4B), slowed infection (Fig 4C), containment (Fig 4D), recurrence (Fig 4E) and clearance (Fig 4F). All other parameters are as in Table 1. (DOCX) [file pcbi.1008451.s019.docx]

| Parameter | No immune response | Widespread infection | Slowed infection | Containment | Recurrence | Clearance |
| --- | --- | --- | --- | --- | --- | --- |
| Virus-receptor association affinity $k_{on}$ (M^-1^ s^-1^) | 1.4×10^4^ | 1.4×10^4^ | 1.4×10^3^ | 1.4×10^2^ | 1.4×10^6^ | 1.4×10^4^ |
| Immune response delay coefficient $\beta_{delay}$ (s) | - | 1.2×10^7^ s | 1.2×10^8^ s | 1.2×10^5^ s | 1.2×10^4^ s | 1.2×10^5^ s |
