# Supplementary material for: A modular framework for multiscale, multicellular, spatiotemporal modeling of acute primary viral infection and immune response in epithelial tissues and its application to drug therapy timing and effectiveness
Source: PLoS Comput Biol. 2020 Dec 21;16(12):e1008451. doi: 10.1371/journal.pcbi.1008451 (PMC7785254; doi:10.1371/journal.pcbi.1008451)
Supplement: S2 Table — (DOCX) [file pcbi.1008451.s020.docx]

| Simulation parameters | Value |
| --- | --- |
| $T_{c}$ formation rate $k_{1}$ | 4800 molecule^-1^ min. ^-1^ |
| Nascent polyprotein cleavage rate $k_{2}$ | 6000 min.^-1^ |
| Viral polyprotein cleavage rate $k_{c}$ | 36 min.^-1^ |
| $R_{P}^{cyt}$ transport rate into cytoplasm $k_{Pin}$ | 12 min.^-1^ |
| $R_{P}$ transport rate into VMS $k_{Pout}$ | 12 min.^-1^ |
| $E^{cyt}$ transport rate in VMS $k_{Ein}$ | 7.8×10-4 min.^-1^ |
| $R_{Ip}$ formation rate $k_{3}$ | 1.2 molecule^-1^ min.^-1^ |
| $R_{P}$ synthesis rate $k_{4p}$ | 102 min.^-1^ |
| $R_{ds}$ synthesis rate $k_{4m}$ | 102 min.^-1^ |
| $R_{Ids}$ formation rate $k_{5}$ | 240 min.^-1^ |
| $R_{P}^{cyt}$ degradation rate $\mu_{P}^{cyt}$ | 600 min.^-1^ |
| $R_{P}$ degradation rate $\mu_{P}$ | 4.2 min.^-1^ |
| $R_{ds}$ degradation rate $\mu_{ds}$ | 3.6 min.^-1^ |
| $R_{Ip}$ degradation rate $\mu_{Ip}$ | 2.4 min.^-1^ |
| $R_{Ids}$ degradation rate $\mu_{Ids}$ | 7.8 min.^-1^ |
| $T_{c}$ degradation rate $\mu_{Tc}$ | 0.9 min.^-1^ |
| $E$ degradation rate $\mu_{E}$ | 2.4 min.^-1^ |
| $E^{cyt}$ degradation rate $\mu_{E}^{cyt}$ | 3.6 min.^-1^ |
| Total number of available ribosomes $R_{ibo}^{tot}$ | 700 |
| RNA conversion factor $n_{HCV}$ | 100 molecule $R$^-1^ |
| $P$ production rate $r_{t}'$ | 2.5 min.^-1^ $R$^-1^ |
