# Supplementary material for: A modular framework for multiscale, multicellular, spatiotemporal modeling of acute primary viral infection and immune response in epithelial tissues and its application to drug therapy timing and effectiveness
Source: PLoS Comput Biol. 2020 Dec 21;16(12):e1008451. doi: 10.1371/journal.pcbi.1008451 (PMC7785254; doi:10.1371/journal.pcbi.1008451)
Supplement: S1 Text — (DOCX) [file pcbi.1008451.s024.docx]

## S1 Text. Integration of an explicit RNA synthesis model

The HCV model in [1] describes subgenomic replication in two compartments, namely the cytoplasm and vesicular membrane structures (VMS). Integration with the viral replication model described in *Quantitative model and implementation* requires the two modifications, one to the HCV model, and one to the viral replication model of the main framework, such that the viral genome taking part in genomic replication from (7) is a proxy for the cytoplasmic plus-strand RNA molecules of the HCV model. Both modifications are described here.

According to the HCV model, in the cytoplasm,

$\frac{dR_{p}^{cyt}}{dt}=k_{2}T_{c}+k_{Pout}R_{P}-k_{I}R_{ibo}R_{P}^{cyt}-k_{Pin}R_{P}^{cyt}-\mu_{P}^{cyt}R_{P}^{cyt}+n_{HCV}r_{u}U,$ (S1)

$\frac{dT_{c}}{dt}=k_{I}R_{ibo}R_{P}^{cyt}-k_{2}T_{c}-\mu_{Tc}T_{c},$ (S2)

$\frac{dP^{cyt}}{dt}=k_{2}T_{c}-k_{c}P^{cyt},$ (S3)

$\frac{dE^{cyt}}{dt}=k_{c}P^{cyt}-k_{Ein}E^{cyt}-\mu_{E}^{cyt}E^{cyt},$ (S4)

where $R_{P}^{cyt}$ is the number of plus-strand HCV RNA molecules in the cytoplasm, $T_{c}$ is the number of translation complexes in the cytoplasm, $P^{cyt}$ is the number of HCV polyprotein molecules in the cytoplasm, $E^{cyt}$ is the number of enzyme NS5B and other viral proteins needed for RNA synthesis in the cytoplasm, $R_{ibo}$ is the number of host cell ribosomes ($R_{ibo}=R_{ibo}^{tot}-T_{c}$ for fixed total available ribosomes $R_{ibo}^{tot}$), and $n_{HCV}$ relates $R_{P}^{cyt}$ to unitless $R$. Simulations of the HCV model were performed as in [1] by initializing $R_{P}^{cyt}$ with an initial nonzero value in the initially infected cell. In the case of a spatial context, where cells are infected at various times according to progression of infection and subsequent internalization events, subgenomic replication within a particular cell occurs due to internalization of virus by the cell (6). As such, the final term of (S1) was added during integration such that internalized virus acts as a source for $R_{P}^{cyt}$.

Likewise, in the VMS,

$\frac{dR_{P}}{dt}=-k_{3}R_{P}E+k_{4p}R_{Ids}+k_{Pin}R_{P}^{cyt}-\left( k_{Pout}+\mu_{P} \right)R_{P},$ (S5)

$\frac{dR_{ds}}{dt}=k_{4m}R_{Ip}+k_{4p}R_{Ids}-k_{5}R_{ds}E-\mu_{ds}R_{ds},$ (S6)

$\frac{dE}{dt}=k_{Ein}E^{cyt}+k_{4m}R_{Ip}+k_{4p}R_{Ids}-k_{3}R_{P}E-k_{5}R_{ds}E-\mu_{E}E,$ (S7)

$\frac{dR_{Ip}}{dt}=k_{3}R_{P}E-k_{4m}R_{Ip}-\mu_{Ip}R_{Ip},$ (S8)

$\frac{dR_{Ids}}{dt}=k_{5}R_{ds}E-k_{4p}R_{Ids}-\mu_{Ids}R_{Ids},$ (S9)

where $R_{P}$ is the number of plus-strand RNA in the VMS, $R_{ds}$ is the number of dsRNA in the VMS, $E$ is the number of HCV polymerase complexes in the VMS, $R_{Ip}$ is the number of plus-strand RNA replicative intermediate complexes in the VMS and $R_{Ids}$ is the number of plus-strand dsRNA replicative intermediate complexes in the VMS.

Having selected $R_{P}^{cyt}$ and $R$ as the shared biological object of the two models, mass action $R\to P$ of the viral replication model of the main framework requires modification. We assume that decay of $R_{P}^{cyt}$ described in the HCV model leads to production of $P$ through intermediate processes. The viral replication model of the main framework ((6)–(10)) then takes the modified form,

$\frac{dU}{dt}=Uptake-r_{u}U,$ (S10)

$n_{HCV}R=R_{P}^{cyt},$ (S11)

$\frac{dP}{dt}=r_{t}'R-r_{p}P,$ (S12)

$\frac{dA}{dt}=r_{p}P-Release,$ (S13)

where $r_{t}'$ is the rate of production of $P$ per unit of $R$ associated with decay of $R_{P}^{cyt}$. Note that without the introduction of additional decay to the HCV model equations, the integrated form of the HCV model effectively acts the same as its original form within each cell, subject to the cellular and spatial aspects of internalization events. All parameters of the integrated HCV model in simulations shown in *Particularization to hepatitis C virus by integration of an explicit RNA synthesis model* were taken from [1] (S2 Table).

References
1. Dahari H, Ribeiro RM, Rice CM, Perelson AS. Mathematical modeling of subgenomic hepatitis C virus replication in Huh-7 cells. J Virol. 2007;81: 750. doi:10.1128/JVI.01304-06
