# Supplementary material for: A modular framework for multiscale, multicellular, spatiotemporal modeling of acute primary viral infection and immune response in epithelial tissues and its application to drug therapy timing and effectiveness
Source: PLoS Comput Biol. 2020 Dec 21;16(12):e1008451. doi: 10.1371/journal.pcbi.1008451 (PMC7785254; doi:10.1371/journal.pcbi.1008451)
Supplement: S2 Text — (DOCX) [file pcbi.1008451.s025.docx]

**S2 Text. Collaborative viral infection modeling environment.**

Given the immense amount of complexity associated with viral infection, supporting collaborative, independent, concurrent, and even conflicting, model development is critical to building an informative and predictive multiscale model of viral infection. As such, the simulation architecture developed for the CompuCell3D implementation, as demonstrated in *Model extensions*, supports development, deployment and distribution of add-on modules following the Python programming language design principles and practices of extensibility and modularity. This architecture exploits the architecture of CompuCell3D itself, specifically, that model implementation in CompuCell3D consists of designing a set of Python classes called “steppables”, each of which is imported into CompuCell3D and simulated (via exactly two simple lines of Python code per steppable, see *S2 Text, Deploying a model extension in CompuCell3D*). Each steppable typically implements a particular model, function, or feature (*e.g.*, viral internalization, data post-processing and exporting), and provides instructions to CompuCell3D about what to do during each simulation step along with the core simulation engine (*e.g.*, implementing the Cellular Potts Model), as well as what to do before and after simulation, through a simple interface (*e.g.*, procedures to perform by a steppable during each simulation step are described in a function “step” in the steppable class definition). This approach is particularly well suited for supporting collaborative, independent, and concurrent model development because model specification of a particular simulation in CompuCell3D consists of selecting and loading a particular set of steppables, each of which can be specified in separate Python scripts and packaged in uniquely named directories, developed by collaborating or independent and otherwise disconnected research groups, and intended to model specific biological phenomena. Furthermore, specification of model implementations using Python classes also enables development of model extensions from existing modules (whether from the main framework or an add-on module) using basic Python class inheritance functionality (where class definitions can be constructed from other class definitions and subsequently modified, see *S2 Text, Extending a model in CompuCell3D*).

We envision a community of modelers much like the community of Python developers, which develops Python packages, called “modules”, that can be publicly distributed and imported into software using a simple, one-line Python command (*e.g.*, import MyModule). As such, we have built into the CompuCell3D implementation used in this work a location for storing a library of add-on modules, as well as supporting architecture to facilitate development and deployment of add-on modules. Furthermore, along with making the simulation framework publicly available online, the online repository also hosts this library of add-on modules as part of the standard download package, which we continue to develop and maintain, and for which we are currently developing standards (*e.g.*, standard documentation) and supporting tools (*e.g.*, documentation generators). We welcome usage by, and contributions from, all interested groups, and provide a basic overview of deploying and developing model extensions in the remaining discussion of this section.

**Deploying a model extension in CompuCell3D**. As in any typical CompuCell3D model specification imports all modules of the main framework and loads them into CompuCell3D for simulation. The directory “Simulation” contains all source code of the main framework, while an additional directory “Models” is dedicated to storing source code of the add-on module library. Each add-on module is an importable Python module stored in its own, uniquely named subdirectory (*e.g.*, Models/IUBIOCAddons). S1 Code Snippet shows a section of the contents of Simulation/ViralInfectionVTM.py for a simulation using the Simple Recovery model described in *An extensible framework architecture enables the inclusion of tissue recovery*.

Lines 1 and 2 in S1 Code Snippet import and load the steppable “ViralInternalizationSteppable” that implements the internalization model described in *E1 - Viral internalization* from Simulation/ViralInfectionVTMSteppables.py. Lines 4 and 5 show that not much is different concerning loading and importing add-on modules. The steppable “SimpleRecoverySteppable” implements the Simple Recovery Model, and is defined in Models/RecoverySimple/RecoverySteppables.py. The only difference between importing and loading a module from the main framework or add-on module library is specifying the location of the Python script containing the steppable to be deployed in a simulation. This way, two model modules can define steppables in Python scripts of the same name without overwriting each other (*e.g.*, Models/GroupX/Steppables.py or Models/GroupY/Steppables.py). The only necessarily unique aspect of a particular model module is the name of its containing directory (*e.g.*, the directory Models/GroupX or Models/GroupY). This scheme isolates model-specific development to the directory in which the add-on model is defined, and modularizes the overall simulation framework into *shareable*, *interchangeable* model components. Furthermore, since development of add-on modules is isolated to a uniquely named directory, the framework promotes concurrent development and implementation of unrelated or even competing models.

**Developing a Model Extension in CompuCell3D**. Developing a model extension is as simple as the typical CompuCell3D model implementation procedure of developing steppables in Python using the CompuCell3D steppable class “SteppableBasePy” (see *S2 Text, Extending a model in CompuCell3D* for discussion of Python class inheritance). S2 Code Snippet shows the application programming interface (API) and select code from the Python script Models/RecoverySimple/RecoverySteppables.py in the add-on module library that implements the Simple Recovery model described in *An extensible framework architecture enables the inclusion of tissue recovery*.

Lines 1-4 in S2 Code Snippet add the directory containing both the main framework and add-on modules library directories using the environment variable “ViralInfectionVTM”, which makes both available to any module loaded into CompuCell3D from Simulation/ViralInfectionVTM.py, whether directly or indirectly (*e.g.*, when Simulation/ViralInfectionVTM.py imports module “A” that imports module “B”). Line 6 imports parameter values of this module defined in Models/RecoverySimple/RecoveryInputs.py, while Line 7 imports a parameter value from the main framework for use in calculations. Line 8 imports the Python standard module “random” for generating random numbers, which, like the rest of the Python standard library and many others, is distributed with CompuCell3D. Line 9 imports everything from the CompuCell3D module “PySteppables”, which contains the available Python classes for Python model implementation in CompuCell3D.

Line 11 in S2 Code Snippet begins the definition of the Simple Recovery model steppable, “SimpleRecoverySteppable”. SimpleRecoverySteppable inherits from “SteppableBasePy”, a steppable class defined in the PySteppables module. Its first three functions, “__init__”, “start”, and “step”, are all functions of the CompuCell3D steppable interface. “__init__” defines the procedures to be performed for initializing the steppable. “start” defines the procedures to be performed after CompuCell3D has initialized but before simulation begins (in the case of this steppable, sharing a reference to itself with the rest of the framework). “step” defines the procedures to be performed during each simulation step (in this case of this steppable, evaluating recovery in each dead cell and executing recovery when it occurs). The final two functions are specific to this steppable. The first, “recover_cell”, performs the necessary procedures associated with recovery on a cell when given one as an argument (*i.e.*, “_cell”). The second, “cell_recovers”, evaluates whether or not a particular cell is recovered. It should be noted that deployment of the SimpleRecoverySteppable class is not limited to usage directly in CompuCell3D as a simulated steppable. Rather, like the importing of a parameter value from the main framework in Line 7 of S2 Code Snippet, the SimpleRecoverySteppable class can be imported into other modules for other purposes, like performing recovery of a dead cell but due to an alternative recovery criterion. The following section describes an example of such functionality.

**Extending a model in CompuCell3D**. Like any other Python class, steppables (and other code) defined in one model module can be extended by, or integrated into, other modules, such that the components of the overall simulation framework are not only interchangeable and shareable, but also extensible. In the previous section, S2 Code Snippet demonstrated the ability to import a parameter value (*i.e.*, “s_to_mcs”, Line 7) from the main framework for usage in an add-on module. The same can be done for integrating modules (whether from the main framework or add-on library) into other add-on modules, as well as for extending model modules using Python class inheritance. Python class inheritance enables the construction of classes from the definition of other classes, such that functionality and interfaces defined by one class can be employed, selectively adapted, and extended, by subsequent classes that inherit from it. Any inheriting class, called a “derived class”, can replace (*i.e.*, “overwrite”) the executed code of a function in the definition of an inherited class, called a “base class”, if the derived class defines a function with the same name and arguments. All inherited functions that are not overwritten by a derived class are the same.

Simulation results of the Neighbor Recovery model in *An extensible framework architecture enables the inclusion of tissue recovery* demonstrate deployment using the framework capability of constructing add-on modules from other add-on modules. Computationally, nearly all aspects of the Recovery Model are the same as the Simple Recovery model (*e.g.*, test for recovery in every cell during each simulation step, implement recovery when it occurs). The only difference between the two models is the criterion by which recovery of a dead cell is evaluated, making the implementation of the Neighbor Recovery model a strong candidate for exploiting Python class inheritance, as demonstrated in S3 Code Snippet.

As in S2 Code Snippet, Lines 1-4 of S3 Code Snippet makes available the entire framework, while Line 5 imports the Simple Recovery model steppable definition for extension. Line 7 begins the definition of the steppable “NeighborRecoverySteppable” that implements the Neighbor Recovery model by inheriting from the class definition for the Simple Recovery model “SimpleRecoverySteppable”. Since all functionality of the Neighbor Recovery steppable is to be the same as the Simple Recovery steppable except for the recovery criterion, Lines 8-9 initialize the Neighbor Recovery steppable exactly the same as the Simple Recovery steppable. Lines 11-14 begin overwriting the definition of the recovery criterion from Simple Recovery according to the Neighbor Recovery model. Since no other functions of the Simple Recovery steppable are overwritten, they are then exactly the same for the Neighbor Recovery steppable. Furthermore, since the signature of the function that implements the recovery criterion (*i.e.*, “cell_recovers(self, _cell)”) has the exact same name and arguments in both steppables (*i.e.*, the function “cell_recovers” is overwritten by NeighborRecoverySteppable), they can be used in the exact same way by other modules (*e.g.*, a variable “my_recovery_steppable”, whether an instance of SimpleRecoverySteppable or NeighborRecoverySteppable, receives and returns the same type of information). The only difference in behavior between using one or the other is the potential outcome of asking either recovery steppable whether or not a particular dead cell recovers, about which the two models will often disagree.
