# Supplementary material for: A modular framework for multiscale, multicellular, spatiotemporal modeling of acute primary viral infection and immune response in epithelial tissues and its application to drug therapy timing and effectiveness
Source: PLoS Comput Biol. 2020 Dec 21;16(12):e1008451. doi: 10.1371/journal.pcbi.1008451 (PMC7785254; doi:10.1371/journal.pcbi.1008451)
Supplement: Attachment — Submitted filename: A Framework for Modeling Infection - Response Letter v1 R1.docx [file pcbi.1008451.s026.docx]

**Reviewer #1**

In this paper the authors address the important issue of viral - immune system interactions within epithelial tissues. The new modelling presented in this paper is distributed per three components, namely: epithelium, ECM, and lymph node component. The open source modelling framework proposed here aims to explore computationally the outcomes of potential treatments.

The novel modelling and computational framework presented in this paper brings a significant contribution towards advancing the understanding of viral infection, and as a consequence I recommend this paper to be accepted for immediate publication in PLOS Computational Biology.

Below, I have a small list of questions and a suggestion regarding the paper structure.

Questions:

Q1: Would it be possible to add more details regarding the way the ECM is accounted for within the model? I am not asking to modify the model, but I feel that the reader would benefit if a paragraph would be added under the Model and Methods section to clarify how the ECM is implicitly/explicitly considered within the model.

**R1.1**. The extracellular matrix (ECM) is of special interest to many fields, and we are grateful to the reviewer for this suggestion. We implicitly represent the effects of the extracellular matrix in the various model parameters related to or affected by it (*e.g.*, viral diffusion coefficient and the motility of immune cells), rather than model it explicitly (though some of us have been working on such a project). We have included some discussion of this in *Models and methods*. See the first paragraphs of *Epithelium component* and *Extracellular environment component*.

Q2: Could the authors comment on how would a heterotypic population of tissue cells make a difference to the modelling (that could perhaps be considered in a future work) and give a hint on what would be a potential impact for the computational results?

**R1.2**. Studying heterotypic cell populations provides a number of excellent opportunities to develop and demonstrate extension to the current framework and their effects! As such, we have added to *Supplementary Materials* a demonstration of employing realistic cell geometries for the epithelial sheet, which showed no significant differences compared to the uniform epithelial sheet used in all other simulations (see Fig S23). Furthermore, we have also added to *Results* a new section on extensions to the models presented in *Models and methods* (see *Model extensions*), one demonstration of which simulates heterotypic populations of varying susceptibility to infection. Specifically, we randomly selected some cells and made them unsusceptible to infection by setting their number of receptors to zero, to model the situation in lungs where different cell types differ in whether they express ACE2, TMPRSS2, etc. To thoroughly investigate the effects of this model extension, we then varied the fraction of the population that are unsusceptible (like studying different locations in the lungs). As expected, having a smaller fraction of infectable cells slows the spread of infection and leads to less structurally organized spread of the lesion (see *Heterogeneous susceptibility inhibits spread of infection*).

Q3: Could the authors add more comments on the perspective of considering more than one generic immune cell type (as this is a heterotypic system) and give a hint on how would that be beneficial to the investigation?

Finally, as a suggestion regarding the paper structure and presentation, it would help the reader if either the entire section Models and Methods (or at least a brief overview of this) would be relocated/placed in the manuscript before the Results section.

**R1.3**. This is a request that we are especially happy to address, as we have been developing a model extension using a heterotypic immune cell population since the initial submission of this manuscript. It is certainly critical to introduce a heterotypic immune cell population to investigate specific spatiotemporal details of the immune response using the framework. We have added to *Discussion* these details, in the second paragraph of *Future perspectives*. Concerning organization of the paper, we appreciate the reviewer’s suggestion on how to better structure the paper, but prefer to follow PLOS policy guidelines on the order of the sections. However, we have made significant revisions throughout the introduction, some of which were made to better facilitate a sense of the modeling work, given the structure of PLOS publications. These revisions can be found in tenth, eleventh and twelfth paragraphs of *Introduction*.

**Reviewer #2**

Sego et al present a computational model of viral infection in an epithelial sheet that includes some aspects of the innate immune response. The model seems to be a reasonable abstraction and qualitatively matches the temporal course of viral infections. As the authors note, its presentation here is to provide an open-source framework for future studies, and as such, it is a useful contribution. In the current manuscript, the key finding cited by the authors is that low levels of inhibition of viral replication, *e.g.*, by a low dose of a drug, can improve infection outcomes more if administered earlier in the progression of viral infection. That is not a surprising finding, but is a reasonable check on the model’s reasonableness. Frankly, I do worry that without a more quantitative alignment with experiments/observations, that the model will not find any more substantial use in understanding infection course or treatment options.

Some specific concerns:

1. The introduction provides a detailed overview of viral infections, including a paragraph on the adaptive immune response that is not actually part of the model. In so doing, the introduction somewhat overpromises in the sense that the model presented later omits many of these details and lumps together others. I would advise the authors to revise the introduction to make clear early on that while the authors are aware of the complexity of the immune response, the model’s abstraction of the immune response will be greatly simplified.

**R2.1**. We are grateful to the reviewer for this feedback. We began building this framework with the immense complexity of the immune system in mind, knowing that much of it is still poorly understood, that modeling all of it one study is likely infeasible (if not too complex to fully appreciate any aspect of it), and that representing it in simulation is of the utmost importance to generating a fully informative and predictive *in silico* representation of events during infection. Hence, the approach that we adopted in this work, namely, to instill a sense of the complexity involved, to represent aspects of that complexity that are critically related to our simulated scenario (and in the simplest terms that we could), and to construct the model modules and computational implementation in such a way as to enable its future development to represent more of that complexity (by us and others, alike). We have revised the introduction throughout to better convey this premise of the work, and to make clear early on what the reader should expect from the models developed in this first work. We have also added a new section to *Results* to demonstrate such activities (see *Model extensions*), as well as an overview of the publicly available architecture and demonstrations of deploying, developing and extending model extensions (see *Appendix S3*).

2. Lines 215-217. The authors note that they “established a baseline set of parameters (Table 1) for which the immune response is strong enough to slow the spread of infection, but insufficient to prevent widespread infection and death of all epithelial cells (Figure 3).” That is a reasonable starting point and the authors later go on to vary some of these parameters by orders of magnitude to map out model time courses across parts of parameter space, but is this baseline parameter set in any sense unique? For example, looking at Figure 5, the results for the baseline parameters (multipliers of 1 and 1) are in that intermediate range, but so are the results for multipliers of 100 and 0.1 (or 0.1 and 100). Why was the {1,1}-multiplier parameter set chosen as baseline rather than one of the other combinations that yielded similar results? Are there other parameter pairs (or triplets) that were not subjected to such large parameter sweeps that could compensate for one another? The authors need to provide more detail/justification for their choice of baseline parameter set.

**R2.2**. Indeed, given the requirements of our baseline parameter set, we could have selected a different set of parameters, and perhaps even more interesting parameters, at that (*e.g.*, a set with more variability in outcomes). Of course, attempting to calibrate the emergent dynamics in simulation to some particular dataset would certainly narrow the available candidates of parameter sets, if not to a unique set, then to a much more limited subset of parameter sets than those available for our requirements in this work. We have added some discussion in the second paragraph of *Results* on the uniqueness of the baseline parameter set.

For our present purpose, we found the logarithmic sweeps to be particularly helpful, and also insightful concerning the emergent dynamics of the underlying complexity, with respect to the variations reported in the initial submission (*e.g.*, virus-receptor association affinity, in Figures 5-7). However, as the reviewer mentions, such variations are not relevant for all parameters. In fact, we have observed much finer sensitivity to other parameters (*e.g.*, viral diffusion coefficient), as well as other instances of the compensation by pairwise variations mentioned by the reviewer (*e.g.*, oxidative death threshold and virally-induced apoptosis dissociation coefficient), in our investigation of the parameter space and determination of our baseline parameter set. We have added to *Supplementary Materials* results from select parameter sweeps to convey these features of the parameter space (Figs S17-S22). In addition we have added a third column to Table 1 in order to describe the justification of each model parameter value.

3. Given the banded nature of the results in Figs 5-10, the complex model constructed by the authors seems to reduce to a comparison of two rates: one related to viral replication and spread; the other related to cell death (both viral and immune-mediated). The authors could increase the paper’s impact by using their model to explore how its parameters impact the relevant rates and the parameter-space locations of the transitions where one rate dominates the other. Helping the community build such intuition would be an excellent use of the model and would increase the likelihood that others would be able to use it effectively in future work.

**R2.3**. We thank the reviewer for this feedback, and for sharing their insight into facilitating broad understanding and utilization of our model in the scientific community. In addition to adding the parameter sweeps in Figs S17-S22 that serve to build a stronger sense of the characteristics of the model parameter space (see **R2.2**), we have revised the manuscript to bring the attention of the reader to these new sweeps when discussing the effects of individual parameters on emergent outcomes according to our classifications defined in *Classification of spatiotemporal infection dynamics*. See the second paragraph of *Discussion*.

4. Lines 677-679. The authors state that “While the simulations presented here begin with a single infected cell, a simulation which begins with multiple infected cells might better represent the infection dynamics of patients that have been subject to high level exposure …” It seems to me that the model may already be simulating a fairly high-level exposure. Since it uses periodic boundary conditions for both the cells and the diffusing fields, I don’t think the model is simulating a single infected cell in a large epithelium, but rather is simulating a case where 1 in 900 cells is infected (ad infinitum repetition of this 900-cell field is implied by the periodic boundary conditions). The authors should explore the literature on viral infections of epithelia and evaluate whether an initial infection of 1 in 900 cells is really a low- or high-level exposure.

**R2.4**. We are grateful to the reviewer for pointing this out. Concerning the conditions of our initial infection, previous works using similar initial conditions, methods of modeling cellular distributions and simulated milieu of infection propagation have indeed employed comparable (but greater) fractions of initially infected cells (Immonen, 2012, 0.5% fraction initially infected; Beauchemin, 2006, 1% fraction initially infected). We have included both of these references with commentary in the second paragraph of *Results*, to facilitate the reader’s sense of how the infection dynamics of our results compare with those previously reported in related works.

5. I appreciate that the authors are making the code for their model open-source, but the “Models and methods” section should still be detailed enough for others to reproduce the model independently. I found the separate presentation of the Conceptual and Quantitative aspects of the model a bit difficult to follow, but that is somewhat a matter of taste. Within the Quantitative sections, I have the following questions / concerns:

**R2.5**. We thank the reviewer for their careful reading of *Models and methods* and we have improved this section according to their feedback. We have responded to their individual comments below.

a. Under E2 – Viral Replication, the description of U as “unpacking” is confusing. “Unpacking” is a process, so it is confusing as to whether U is the amount of virus that has been unpacked or the amount that has not yet been unpacked. Given the structure of Eqn 6, I suspect it is the latter, since dU/dt increases as Uptake increases and decreases in proportion to the amount of U currently in existence. This should be clarified as should all the descriptions in that section. Each of the variables U, R, P and A should be explicitly described as the amount of some object/molecule rather than as a process. The time derivatives are the process.

**R2.5.a**. We agree with the reviewer and have renamed the variables U (internalized virus), R (viral genome taking part in genomic replication), P (synthesized protein) and A (assembled and packaged virions) and to represent a measurable amount while keeping the existing names for the processes themselves. See the first paragraph of *E2 - Viral Replication*.

b. Line 945. Isn’t the replication rate reduced to (rmax/2)R rather than just rmax/2.

**R2.5.b**. The flux of replication is reduced to (rmax/2)R while the rate is reduced to rmax/2. We have revised the text to make this clearer.

c. Under E4 – Cell death. What happens to internal assembled virus upon virus- or immune-induced cell death? Are these assembled viruses released or destroyed in the model?

**R2.5.c**. We assume that in the process of death the assembled virus and viral particles would be either damaged or deactivated. We have added to the end of *E4 – Cell death* an explicit statement of this assumption.

d. Why were all the Hill factors chosen to be 2? Why is a Hill factor not listed as a parameter in Eqn 21 and is instead simply designated as 2?

**R2.5.d**.  We made the decision to use the standard Hill factor of 2 to represent the activation of our various processes using a smooth function like simple cooperativity. Similar qualitative results are seen for different Hill factors but for much larger than 2 start to approximate a simple step behavior, and this is not what we are modelling. We have listed the Hill factor of Equation (21) as a parameter now in Table 1.

e. The authors should carefully check all their del and gradient symbols. Some are inverted from the others and it is not clear that this is intentional.

**R2.5.e**. We have revised *Models and methods* throughout for consistent usage of deltas and nablas. See Equations (20), (21) and (23).

f. Section I3 – Immune cell direct cytotoxicity and bystander effect. This short section is very unclear. I cannot tell what is actually coded.

**R2.5.f**. We have revised *I3 – Immune cell direct cytotoxicity and bystander effect* to improve overall clarity and include sufficient mathematical detail to convey the calculations of direct cytotoxicity and bystander effect killing.

g. Section I4 is missing any math details.

**R2.5.g**. We have added a formal mathematical expression to this section for killing by immune cell oxidizing agent cytotoxicity. See Equation (19).

h. Section T2- Cytokine transport. Eqn 21 is presented with a very general c_sig that is only described as “a quantity that affects its cytokine secretion.” This initially left me very confused until later in the paragraph where c_sig is noted to be different quantities in different situations (amount of assembled virus or local cytokine concentration). Given that these are two very different means of triggering cytokine secretion, why do they both have the same dissociation coefficient V_cyt?

**R2.5.h**. Vcyt is not the same value or quantity for epithelial / immune cells, which was not adequately described in the initial submission. For epithelial cells, Vcyt is the amount of internally assembled virus, while for the immune cells, Vcyt is the amount of cytokine the cell is exposed to. We have revised this section to make this distinction clear. See *T2 – Cytokine transport*.

i. In the table of baseline parameter values (Table 1), the formatting of some entries is ambiguous, e.g., is 1/6x10^(-3) really 0.167 x 10^(-3) or 1/(6x10^(-3))? And towards the end of the table, there are several entries where items that should be superscripts are not.

**R2.5.i**. When we had written 1/6x10^(-3), we were meaning (1/6)x10^(-3). We have revised Table 1 to make this clear. We have also checked all superscripts of Table 1 in our revision. As discussed in **R2.2**, we have also extended the parameter table to include sources or justifications of all parameters. See Table 1.

**Reviewer #3**

In this article, the authors present a multiscale, multicellular computational framework to model the spatio-temporal dynamics of viral spread and immune responses during acute infection. Analyzing different scenarios they use their model to investigate the effect of different drug therapy regimens on disease outcome.

The paper is well written and the structure of the modeling framework is explained. However, in my view, the presented results do not provide novel insights for understanding viral infections or drug therapy in general or for specific viruses, nor do they emphasize the necessity to consider spatio-temporal dynamics when analyzing viral spread. Using the model to study specific viral infections would also require careful parameterizations and adaption of a lot of aspects of this model to this particular virus so that it is not clear how this would provide a general framework for analyzing viral spread in its current stage.

\# Major points:

(1.) The simulation environment is quite complex including a lot of different factors and processes to capture relevant aspects during acute infection. In order to get meaningful results and insights, parameterization of these processes according to experimental or clinical data is the key, and the most difficult part. For most of the parameters, and especially the virus related ones, no sources for the choice of parameters are given (Table 1). It is not clear if the model was parameterized considering a specific virus but the later statements (line 657-658) indicate that this is only work in progress. It is questionable how meaningful the results are if arbitrary values were chosen, even if they were used for qualitative comparisons.

**R3.1**. We thank the reviewer for the feedback and we realize that the parameter table did not sufficiently explain the parameters. As discussed in **R2.2** we have added an extra column to the table called “References / Justification” where we either cite a source or give a short justification for each parameter. See Table 1. Concerning the virus of our simulations, we parameterized the viral replication model and related mechanisms according to reported data on the timescale of progression of infection in SARS-CoV-2. We have included this in our new column of Table 1 and explicitly stated this in the second paragraph of *Results*.

(2.) Although modeling spatio-temporal dynamics of viral spread, the analysis is done on a simple squared lattice of epithelial cells. To make an argument for considering spatial aspects, the authors need to investigate how much the results would differ if they would use population-based ODE-models instead (as they acknowledge themselves, line 598-604). Without investigating processes that specifically imply considering spatial aspects (tissue regeneration, cell-transmission modes, local immune responses) the complexity of the model would not necessarily provide additional insights.

**R3.2**. We thank the reviewer for this feedback. Certainly, there are differences in predicted dynamics and outcomes when comparing results from population-based ODE models and spatial, stochastic, cell-based models like ours (as each explicitly or implicitly describes different biophysical objects). In our revision of the introduction, we have added a survey of such notable differences as relevant to the scope of our work. In particular, spatial models produce significantly different viral and immune dynamics alike, as well as parameter estimates and therapeutic predictions. We included references to differences in predictions of the magnitude and time of peak viral loads, as well as to works related to important predictions of infectivity, viral diffusion, and the basic reproductive ratio, that stochastic, spatial models like ours may be better suited to make (*e.g.*, modeling the effects of local availability of target cells, or local virus diffusivity). We have added this survey (as well as other related content, see **R3.3**) to the third, fourth and fifths paragraphs of *Introduction*.

Of course, as the reviewer points out, a reader who is not interested in investigating, say, local immune responses or tissue regeneration, may not be particularly interested in our model’s capabilities concerning local immune response or developing extensions of tissue recovery like those featured in our new subsection *An extensible framework architecture enables the inclusion of tissue recovery*. While we believe this to be an appropriate limitation of our model (or rather, of any model), we also believe that there is value in our work in the capability to investigate the cellular and spatial processes that lead to emergent, aggregated outcomes like those described by ODE models of population dynamics. Furthermore, we also believe that there is value in our work in the capability to investigate subcellular processes of particular viral infections, and models thereof, in a multicellular, spatial context (as demonstrated in the new section entitled *Integration of an explicit RNA synthesis model allows the spatiotemporal modelling of hepatitis C virus infection*, see **R3.4**). We have added commentary to this effect in the fifth paragraph of Discussion.

Concerning the simplified geometry of our square lattice, along with the discussion already included in *Future perspectives* on applying our framework to the problem of multi-organ disease progression (third paragraph), we have added new content that demonstrates elementary steps towards applying our framework to advanced tissue structures, of which many comparable examples using the Cellular Potts model and CompuCell3D can be found in the literature (and is well demonstrated in Swat, 2012). In particular, we have added demonstrations of progression of infection in an epithelial sheet of non-uniform cell geometries and heterogeneous cell populations (see **R1.2,** Fig S23 and *Heterogeneous susceptibility inhibits spread of infection*), as well as a note to the reader in the first paragraph of *Future perspectives* that extending the framework to more advanced geometries is easy but, as one might expect, comes at the expense of increased computational cost.

(3.) There have been a number of reviews that address the multi-scale modeling of viral spread and immune responses, and when it is necessary to consider spatial aspects (e.g. Bauer et al. Inf Sciences 2009, Graw & Perelson Annals of Virology 2016). They cite several studies that have used spatially-resolved multi-scale models for different viruses.

**R3.3**. We are grateful to the reviewer for this feedback on improving the broader context of our work in the literature, and especially for providing specific examples. In our revision of the introduction, we have added a significant amount of new content describing related works in the literature, ranging from compartmental ODE models, to spatial and agent-based models, with a special emphasis on differences in predictions between those generated by ODE models, and those generated by spatial and agent-based models (as described in **R3.2**). We have also highlighted works in the literature related specifically to important observations from our results concerning the significant effects of stochasticity early in infection (as described in *Even moderate inhibition of genomic replication by antiviral therapies significantly reduces the spread of infection, but only when initiated soon after infection*), when heterogeneous cell distributions and cell-based models like ours are particularly well-suited for modeling and simulation. This new content can be found in the second, third, fourth and fifth paragraphs of *Introduction*.

(4.) Many viruses (HIV, HCV, Influenza A, ..) have different replication mechanisms, target tissues and relevant immune responses. That means, despite providing a modeling framework, many aspects of the model need to be adapted to investigate the spatio-temporal dynamics for a particular virus. It is not clear how flexible the modeling framework is for this task.

**R3.4**. We thank the reviewer for this feedback, and agree that the adaptability and extensibility of the framework was not sufficiently described in the initial submission. As such, we have put forth a major effort to describe and demonstrate these aspects, both in application and in procedure. Specifically, we have added five new subsections to the manuscript.

In the main body, we have added a new subsection to *Results* entitled *Model extensions*, which presents three primary activities of what we envision would be done with our framework upon broad scientific use. First of these, the subsection entitled *Heterogeneous susceptibility inhibits spread of infection* demonstrates basic extension of the model to particularize the simulated tissue to a specific location of the lungs by introducing heterogeneity to the epithelial sheet, as suggested by Reviewer 1 (see **R1.2**). Second of *Model extensions*, the subsection entitled *Integration of an explicit RNA synthesis model allows the spatiotemporal modelling of hepatitis C virus infection* demonstrates integration of an existing, explicit model of subgenomic replication of hepatitis C (thanks to the reviewer for the idea in this comment), which both particularizes our framework to another virus (a critical procedure when using our framework) and allows the study of an existing ODE model in the context of spatiotemporal infection events (a critical capability enabled by our framework). Third of *Model extensions*, the subsection entitled *An extensible framework architecture enables the inclusion of tissue recovery* demonstrates both adding a model extension to the framework, and also adding an extension of a model extension, through simple examples of modeling tissue recovery (thanks again to the reviewer for the idea in their first comment). Both of the procedures demonstrated in this third new subsection are critically important to supporting continuous improvement of the framework and its utilization by various groups with even conflicting ideas about how to model various biophysical mechanisms.

The final two new subsections were added to *Supplementary Materials*. The first of these, *Appendix S2*, complements our new subsection in the main body on the integrated model of subgenomic hepatitis C virus replication by describing the basic biological premise and mathematical details of the integration procedure. The last of the new subsections, *Appendix S3*, describes the scope of the framework software and its usage and development by the scientific community, which we publicly maintain on GitHub specifically to enable community-driven development, collaboration and model sharing. This new subsection, which we reserved for the supplementary materials only to prevent distraction (if not dissuasion) of non-programmers by featured code snippets, describes the details of the built-in software modularity and extensibility, which were designed using these same principles that drive much of the popularity of the Python programming language (in fact, add-on modules to our framework are developed, packaged, distributed and used in the same way as Python modules). We demonstrate to the reader all fundamental aspects of the software relevant to these concepts, from how to use publicly available model extensions in their own simulations, to how to develop and add their own model extension to the public library of modules, to how to integrate and extend available model extensions and the modules of Models and methods in their own model extensions. To facilitate developing an intuition of the purpose and outcomes of these procedures, we generated the walk-throughs of this new subsection with supporting code snippets from the source code of the extension demonstrations featured in *An extensible framework architecture enables the inclusion of tissue recovery*.

(5.) The aspects of time of treatment and drug effectiveness have been investigated using ODE-models, even for SARS-CoV-2. The studies come to similar conclusions without the need to consider spatial aspects and by analyzing clinical data, i.e., obtaining relevant parameterizations (Goncalves et al. CPT Pharmacometrics 2020, Goyal et al. medRxiv 2020).

**R3.5**. We are grateful to the reviewer for sharing this new published work (which appeared after submitting our manuscript) and preprint. We are also encouraged to see agreement between our model predictions about early treatment and those of an ODE model, which certainly adds to the seriousness with which the prediction should be taken by the scientific and medical communities. This is especially true since the premise of the published model by Gonçalves et. al. and their method of modeling therapeutic intervention are different. Namely, the work by Gonçalves et. al. present a deterministic, non-spatial ODE model of population dynamics and viral load and implicitly model a mode of action (though not necessarily so) through the reduction of the basic reproduction number, while we modeled our intervention as explicitly affecting a particular mechanism of viral infection in a spatial context and with sufficient stochasticity to enable the prediction of uncertain outcomes (another difference between the two models). Probably most exciting to us is the potential to use these ODE models in tandem with ours, where the ODE model forms and calibrated parameters can be employed in our framework when particularizing it further for SARS-CoV-2 (we are currently writing about a method to relate information between spatial and ODE models), and our model can inform these ODE models about the effects of stochasticity, spatial mechanisms, and locale-specific dynamics. In fact, since our initial submission of this manuscript we have generated instances of our framework using the models in both Gonçalves et. al. and Goyal et al. We have added these details to the manuscript, in the fourth paragraph of *Discussion*, and the fourth and fifth paragraphs of *Future Perspectives*.

\# Minor points:

- The model and the analysis itself (tissue structure, parameterization, pharmacodynamics) is not COVID-specific. Therefore, using COVID-19 as a keyword or prominent “example” is misleading.

**R3.6**. We thank the reviewer for this warning, and have revisited the appropriateness of using COVID-19 as a keyword for the work presented in this manuscript. While there are many aspects of the results that could be argued to implicitly relate the work to SARS-CoV-2 (*e.g.*, variability in outcomes demonstrated throughout *Results*, albeit not uniquely so), we feel that the connection is appropriate for two reasons. First, the drug scenario presented in *Even moderate inhibition of genomic replication by antiviral therapies significantly reduces the spread of infection, but only when initiated soon after infection* simulates the mode of action of drugs like remdesivir, which is currently under evaluation for treatment of COVID-19. By categorizing this work as related to COVID-19, we can bring the attention of those investigating various aspects of remdesivir (and other similar therapies) to both the predictions of our scenario (and, as the reviewer mentioned previously, the same prediction of our colleagues developing ODE models), as well as the modeling capability of our framework. Second, to improve the relevance of this scenario (and of our simulations, in general), we calibrated the parameters of our viral replication model with reported time scales for SARS-CoV-2. This was not explicitly stated in the initial submission. We have stated this in our revision, in the second paragraph of *Results*.
